# The evolutionary patterns, expression profiles, and genetic diversity of expanded genes in barley

**DOI:** 10.3389/fpls.2023.1168124

**Published:** 2023-04-26

**Authors:** Wenjing Tao, Ruiying Li, Tingting Li, Zhimin Li, Yihan Li, Licao Cui

**Affiliations:** ^1^College of Bioscience and Engineering, Jiangxi Agricultural University, Nanchang, Jiangxi, China; ^2^State Key Laboratory of Cellular Stress Biology, School of Life Sciences, Faculty of Medicine and Life Sciences, Xiamen University, Xiamen, Fujian, China

**Keywords:** barley, gene family expansion, gene duplication, evolutionary rate, expression pattern, genetic diversity

## Abstract

Gene duplication resulting from whole-genome duplication (WGD), small-scale duplication (SSD), or unequal hybridization plays an important role in the expansion of gene families. Gene family expansion can also mediate species formation and adaptive evolution. Barley (*Hordeum vulgare*) is the world’s fourth largest cereal crop, and it contains valuable genetic resources due to its ability to tolerate various types of environmental stress. In this study, 27,438 orthogroups in the genomes of seven Poaceae were identified, and 214 of them were significantly expanded in barley. The evolutionary rates, gene properties, expression profiles, and nucleotide diversity between expanded and non-expanded genes were compared. Expanded genes evolved more rapidly and experienced lower negative selection. Expanded genes, including their exons and introns, were shorter, they had fewer exons, their GC content was lower, and their first exons were longer compared with non-expanded genes. Codon usage bias was also lower for expanded genes than for non-expanded genes; the expression levels of expanded genes were lower than those of non-expanded genes, and the expression of expanded genes showed higher tissue specificity than that of non-expanded genes. Several stress-response-related genes/gene families were identified, and these genes could be used to breed barley plants with greater resistance to environmental stress. Overall, our analysis revealed evolutionary, structural, and functional differences between expanded and non-expanded genes in barley. Additional studies are needed to clarify the functions of the candidate genes identified in our study and evaluate their utility for breeding barley plants with greater stress resistance.

## Introduction

Gene duplication provides a rich source of genetic material that facilitates genome evolution and environmental adaptation; it is thus an important driver of genomic and genetic diversity (Kimura and Ohta, 1974; Panchy et al., 2016). Due to whole-genome duplication (WGD) and small-scale duplication (SSD) events, plant genomes contain a large number of duplicated genes (Freeling, 2009; Glover et al., 2015). WGD occurs frequently in the plant kingdom but rarely in the animal and fungal kingdoms (Salman-Minkov et al., 2016). WGD events have been documented in multiple angiosperms, such as rice (Yu et al., 2005), maize (Gaut et al., 2000), and cotton (Li et al., 2015; Wang et al., 2022). SSDs, including tandem, segmental, and transposon-mediated duplications, are more common in plant genomes (Gout and Lynch, 2015; Panchy et al., 2016).

Thousands of duplicated genes have accumulated deleterious mutations over the evolutionary history of plants, and this has led to the generation of pseudogenes and eventually gene loss (Lynch and Conery, 2000; Assis and Bachtrog, 2013). However, many gene duplicates are preserved in the genome due to neofunctionalization, subfunctionalization, and increased gene-dosage advantage. In the neofunctionalization model, a copy maintains its ancestral function under negative selection (also known as purifying selection), and the new copy evolves under positive selection due to the generation of adaptive mutations (Walsh, 1995; Pegueroles et al., 2013). For example, aspen *FD* genes have evolved to transcriptionally regulate adaptive responses and the maturation of buds rather than interact with FLOWERING LOCUS T (FT) protein (Tylewicz et al., 2015). In the subfunctionalization model, two copies perform complementary functions and accumulate mutations, and their rates of evolution increase symmetrically under negative selection (Lynch and Force, 2000; Pegueroles et al., 2013). A classical duplicated gene of the anthocyanin biosynthetic pathway in *Ipomoea* can be explained by the subfunctionalization model (Des Marais and Rausher, 2008). Moreover, two cation/proton antiporter 1 (CPA1) protein family members in grapevine, *VIT_19s0090g01480* and *VIT_05s0020g01960*, have undergone subfunctionalization, and this has mediated the response to salt stress in different tissues and stages (Ma et al., 2015). In the dosage model, all duplicated genes increase the quantity of protein products, and they are rapidly fixed under positive selection (De La Torre et al., 2015). In *Arabidopsis thaliana*, the genes involved in glycolysis have duplicated, and this has enhanced energy production (Blanc and Wolfe, 2004).

A gene family is a group of paralogous genes produced by gene duplication that usually show structural and functional similarities (Tatusov et al., 1997). In recent years, an increasing number of studies have shown that expansions of certain gene families, caused by duplication, are associated with specific traits, resistance to environmental stress, or adaptation in plants. Expanded gene families in jackfruit are involved in the response to biotic stimuli, transferase activity, and oxidoreductase activity (Lin X. et al., 2022). *KASI* and *SAD* genes have expanded in the macadamia genome and are involved in the elongation of fatty acid chains (Lin J. et al., 2022). Expansion of the chalcone synthase (*CHS*) gene family in mango has mediated the biosynthesis of urushiols and related phenolics (Wang et al., 2020). WGD and polyploidization events in elephant grass have increased the number of genes involved in rapid growth, biomass accumulation, and drought tolerance (Zhang et al., 2022). Expanded gene families provide a rich resource for functional research and genetic breeding in crops.

Barley (*Hordeum vulgare*) was domesticated in the Fertile Crescent approximately 10,000 years ago, making it one of the world’s earliest domesticated crops (Ullrich, 2010; Zeng et al., 2018). Today, barley is the fourth most produced grain globally after maize, rice, and wheat, and it is widely used in brewing, feed, food, and medicine (https://www.fao.org/). Barley is a highly adaptable crop compared with wheat; it is known to be highly resistant to high salinity and can be grown at high altitudes (Nevo et al., 2012). The newly updated barley genome assembly (Morex V3) is the most complete characterization of the barley genome to date, and this has accelerated comparative genomics analyses of barley and other species (Mascher et al., 2021). Here, proteins from barley and six other Poaceae species were found to be clustered in 27,438 orthogroups, and 214 have undergone significant expansions in barley. The non-synonymous substitution rate (Ka), synonymous substitution rate (Ks), and Ka/Ks were calculated to assess the evolutionary rates of and selection pressures on expanded and non-expanded genes. We found that the expanded genes are evolving faster, are smaller, show weaker codon usage bias, are more weakly expressed, and show higher tissue specificity in their expression than non-expanded genes. Our data also indicate that the expanded genes were involved in responses to biotic and abiotic stresses. Overall, our study reveals the evolutionary trajectories and roles of expanded genes in barley and provides new genetic resources that will aid subsequent functional studies and the breeding of improved barley varieties.

## Materials and methods

### Gene family expansion/contraction analysis

Non-redundant protein sequences from barley (Morex V3 HC protein) and six other Poaceae species were obtained, including *Brachypodium distachyon* (Brachypodium_distachyon_v3.0), rice (*Oryza sativa*, MSUv7), sorghum (*Sorghum bicolor*, Sorghum_bicolor_NCBIv3), rye (*Secale cereale*, Rye_Lo7_2018_v1p1p1), foxtail millet (*Setaria italica*, Setaria italica v2.0), and maize (*Zea mays*, Zm-B73-REFERENCE-NAM-5.0). OrthoFinder v2.5.4 was used for orthogroup clustering with the following parameters “-M msa -S diamond” (Emms and Kelly, 2019). Orthogroups containing more than 100 genes were not preserved for subsequent analysis. An ultrametric tree was constructed using the R8s program and a phylogenetic tree generated from 5,635 single-copy orthologs (Sanderson, 2003). The calibration time between barley and rice (median time = 50 Mya) was queried from the TimeTree database (http://timetree.org) (Kumar et al., 2017). Gene family expansion and contraction were determined by CAFÉ v4.2, and the threshold for statistical significance was *p*-value < 0.05 (De Bie et al., 2006). Syntenic analysis was performed to reveal the duplication mechanism of the expanded genes using BLASTP and MCscanX software with default parameter (Wang et al., 2012). Transposable element (TE) annotation file was downloaded from e!DAL database (https://doi.ipk-gatersleben.de/DOI/b2f47dfb-47ff-4114-89ae-bad8dcc515a1/865cd721-0571-473b-a2ec-92ce51ded713/0). Overlap analysis between expanded genes and TEs was carried out using bedtools v2.28.0. A search for the nucleotide binding-site–leucine-rich repeat (NBS-LRR) gene family was performed using InterProScan v5.56-89.0 against the Pfam NB-ARC domains (PF00931) (Blum et al., 2021). The chromosome locations of *NBS-LRR* genes were visualized using MapChart v2.32 (Voorrips, 2002).

### Estimation of substitution rates

To obtain a more comprehensive picture of gene–pair relationships, we compared barley with rye and *B. distachyon*. Multiple sequence alignment was performed using Clustal v1.2.4 (Higgins and Sharp, 1988). The PAL2NAL program (http://www.bork.embl.de/pal2nal/) was used to convert amino acid alignments into codon alignments (Suyama et al., 2006). The Ka, Ks, and Ka/Ks values were calculated by the CODEML sub-program in PAML v4.9 (Yang, 2007). Homologous gene pairs with Ka > 2, Ks > 2, Ks < 0.01, and Ka/Ks >10 were discarded, as these abnormal values can result in inaccurate estimates or the saturation of substitutions (Villanueva-Cañas et al., 2013).

### Characterization of gene structure and codon usage bias

The generic feature format file of barley Morex V3 (http://doi.org/10.5447/ipk/2021/3) and an in-house python script were used to calculate the gene length, intron length, exon length, first exon length, and number of exons (Mascher et al., 2021). Proteins with sequences longer than 100 amino acids were included to estimate codon usage bias. The codon adaptation index (CAI), codon bias index (CBI), frequency of optimal codons (Fop), and GC content were computed using CodonW v1.4.4 (http://codonw.sourceforge.net/).

### Functional enrichment analysis and transcription factor identification

EggNOG-mapper v2.1.7 (http://eggnog-mapper.embl.de/) was used to assign Gene Ontology (GO) and Kyoto Encyclopedia of Genes and Genomes (KEGG) annotations (Huerta-Cepas et al., 2018; Cantalapiedra et al., 2021). GO and KEGG enrichment analyses were performed using Tbtools v1.098763 (Chen et al., 2020). Plant Ontology (PO) enrichment analysis was carried out using the enricher function in the clusterProfiler package (Wu et al., 2021). GO terms, KEGG pathways, and PO terms with *p*-values < 0.05 and corrected *p*-values (Benjamini and Hochberg method) < 0.05 were retained. Transcription factors (TFs) were identified using the Plant Transcription Factor Database (PlantTFDB v5.0, http://planttfdb.gao-lab.org/prediction.php).

### Expression profiling and co-expression network analysis

A total of 174 RNA-seq samples from 16 tissues/stages (PRJEB14349) and under different abiotic stresses (heat, salt, waterlogging, and water-deficit stress) (PRJNA32416, PRJNA54259, PRJNA602700, and PRJNA439267) were downloaded from the National Center for Biotechnology Information (NCBI) Sequence Reading Archive (SRA) database. Detailed sample information is provided in **Supplementary Table S1**. Raw reads were preprocessed using Trimmomatic v0.36 (Bolger et al., 2014). The high-quality reads were mapped to the barley reference genome (Morex V3) with HISAT v2.1.0 (Kim et al., 2015). SAMtools v1.3.1 was used to sort BAM files (Li et al., 2009). Fragments per kilobase of exon per million fragments mapped (FPKM) values were calculated using StringTie v1.3.5 with the genomic annotation file (Pertea et al., 2015). Highly and weakly expressed genes were genes with FPKM ≥ 50 and FPKM ≤ 3, respectively (Chen et al., 2013; Guo et al., 2017). The value of τ varies from 0 to 1 and was used to measure tissue specificity, with higher τ values implying higher tissue specificity (Yanai et al., 2004). Categorical and overall tissue-specific genes refer to genes expressed in one tissue (also defined as τ = 1) and two or more tissues (also defined as τ < 1), respectively (Schug et al., 2005).

Weighted correlation network analysis (WGCNA) was employed to construct co-expression networks using the FPKM of the coding sequences (CDSs). The numbers 6 and 20 were the soft-thresholding powers for stage/tissue and stress networks, respectively. The co-expression modules were obtained using the parameters “mergeCutHeight = 0.2 and minModuleSize = 30.” The top 1% weighted values associated with TF-type expanded genes were retained for subsequent analysis (Langfelder and Horvath, 2008). The BLASTP search of barley and *A. thaliana* (https://www.arabidopsis.org/) predicted similar proteins and potential functions of co-expressed genes with an *E*-value < 1e-5. Cytoscape v3.7.2 was used to visualize the co-expression networks. For genes with homologs in *A. thaliana*, the gene IDs of barley were annotated as gene IDs in *A. thaliana* to more visually reflect the potential biological functions of the genes (Shannon et al., 2003).

### Read mapping and nucleotide variant calling

The barley resequencing datasets were retrieved from the NCBI SRA database (PRJEB8044), including 89 wild accessions and 126 landrace accessions (Russell et al., 2016). The detailed information is shown in **Supplementary Table S2**. Trimmomatic v0.36 was used to perform quality control (Bolger et al., 2014). BWA v0.7.17-r1188 was applied to construct the genome index of barley Morex V3 and map the clean reads to the reference genome using the BWA-MEM algorithm (https://github.com/lh3/bwa). Picard tools v2.1.1 was used to sort the BAM files and remove duplicates induced by PCR amplification (https://broadinstitute.github.io/picard/). SNP calling was performed by the HaplotypeCaller module in GATK v3.5 (https://github.com/broadinstitute/gatk). The SNPs were filtered using the following criteria: quality by depth (QD) < 2.0, Fisher strand (FS) > 60.0, mapping quality (MQ) < 40.0, mapping quality rank sum (MQRankSum) < –12.5, and read position rank sum (ReadPosRankSum) < –8.0. The SNPs were annotated using SnpEff v5.1d with the parameters “-no-intergenic -no-downstream -no-upstream” (Cingolani et al., 2012). SNPs with minor allele frequency (MAF) > 0.05 and max missing rate < 0.2 were retained for subsequent analyses.

### Population genetics and haplotype analysis

Principal component analysis (PCA) was performed using the smartPCA algorithm in the EIGENSOFT v7.2.1 program (Price et al., 2006). The Tracy–Widom test was used to determine the significance of the eigenvectors. The neighbor-joining (NJ) tree was generated using TreeBest v1.9.2 with 1,000 bootstrap replicates. FigTree v1.4.4 was used to visualize the phylogenetic tree (http://tree.bio.ed.ac.uk/software/) (Vilella et al., 2009). Population structure was analyzed using ADMIXTURE v1.3.0 with K-values from 2 to 4 (Alexander et al., 2009). Nucleotide diversity (π) and Wright’s F-statistic index (*F_ST_*) were estimated using VCFtools v0.1.16 (Danecek et al., 2011). DnaSP v6.12.03 was used to identify haplotypes, and the haplotype networks were visualized using PopART v1.7 with the median-joining method (Bandelt et al., 1999; Leigh and Bryant, 2015; Rozas et al., 2017). The online Gene Structure Display Server v2.0 (http://gsds.gao-lab.org/) was used to visualize gene structure and SNP locations (Hu et al., 2015).

### Plotting and statistical tests

**Figure 1:**
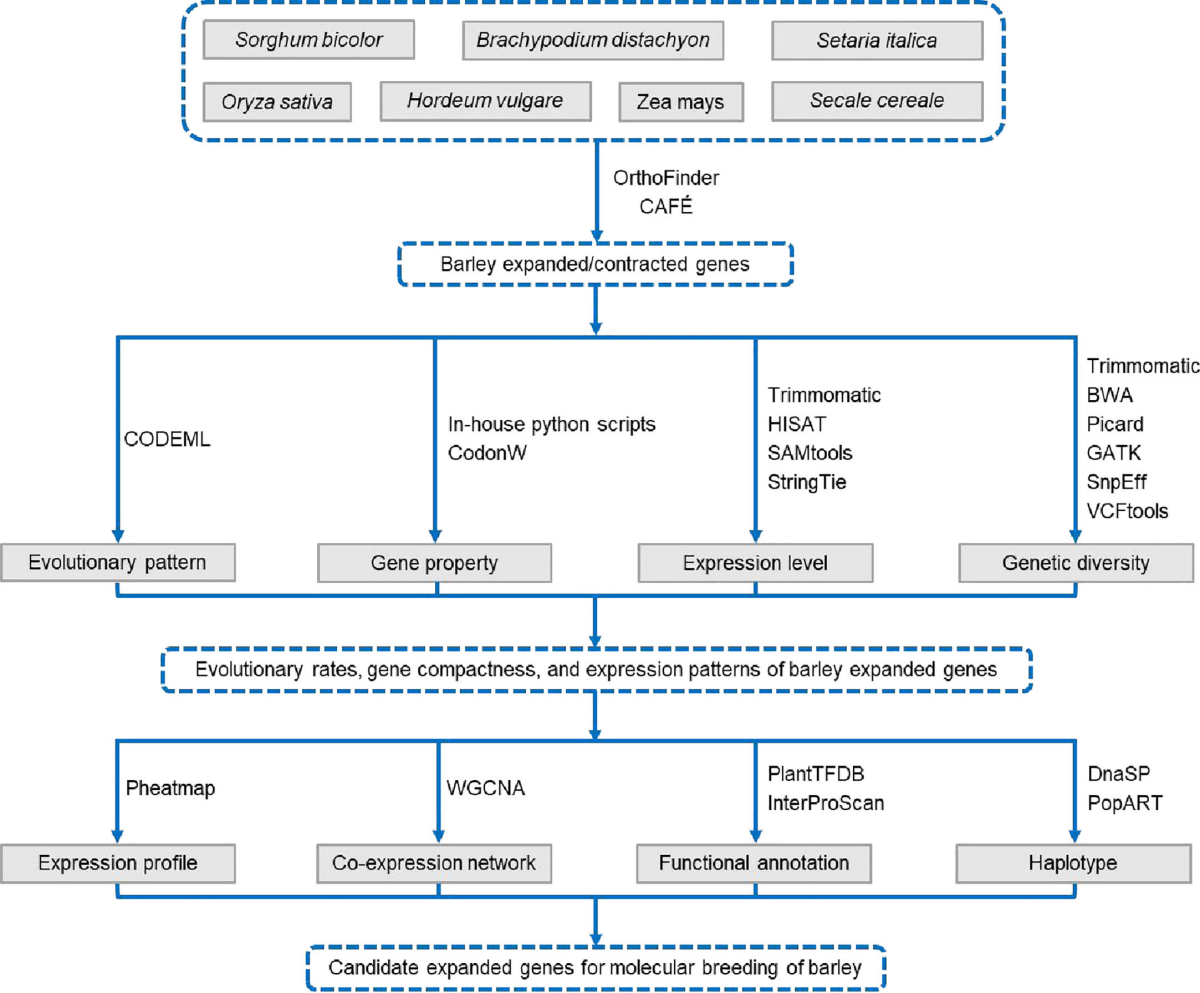
Workflow diagram.

The R (v4.1.0) package ggplot2 was used to generate stacked plots, frequency distribution plots, and box plots, and the plots were integrated into panels using the cowplot package. The RIdeogram package was used to visualize the chromosomal distributions of the Ka, Ks, and Ka/Ks values. Heat maps were drawn using the pheatmap package. The correlation heatmaps were visualized using the corplot package. The wilcox.test, cor.test, chisq.test, and LSD.test functions in the R statistical environment were used to perform the Mann–Whitney U test, Spearman’s rank correlation test, Fisher’s exact test, and least significant difference (LSD) test, respectively. The three levels of significance in all statistical tests were as follows: *p < 0.05, **p < 0.01, and ***p < 0.001. The workflow is shown in **Figure 1**.

## Results

### Homologous clustering and gene family expansion/contraction analysis

**Figure 2:**
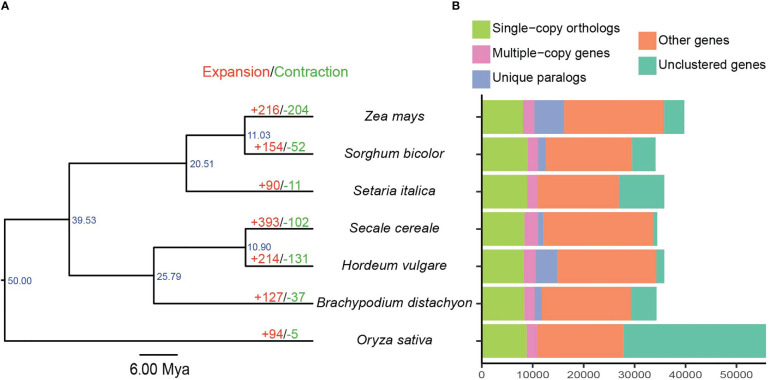
Gene clustering and species phylogenetic analysis. **(A)** Phylogenetic tree, divergence time, and gene family expansions/contractions among seven Poaceae species. Scale bar corresponds to 6 Mya. **(B)** Distribution of homologous genes.

A total of 217,329 proteins from seven related genomes were clustered into 27,438 orthogroups (**Supplementary Tables S3**, **S4**). The protein-coding genes of barley were assigned to 19,768 orthogroups with an average of 1.73 genes per group, and 398 orthogroups were unique to barley (**Supplementary Table S4**). A phylogenetic tree was constructed using 5,635 single-copy orthologous genes from these seven species (**Figure 2A**). The fossil-calibrated phylogenetic tree revealed that barley was phylogenetically closely related to rye (10.90 Mya) and *B. distachyon* (25.79 Mya), and rice, an outgroup taxon, was phylogenetically distant from the other six species. Moreover, maize, sorghum, and foxtail millet were clustered into one clade, which was consistent with the results of a previous study (Li G. et al., 2021). Gene family expansion/contraction analysis revealed 214 expanded, 131 contracted, and 17,546 non-expanded orthogroups in barley (**Figure 2B**). We ultimately identified 3,496 expanded genes and 21,537 non-expanded genes in barley.

Syntenic analysis was performed to elucidate the possible origin of the expanded genes. A total of 2,428 (69.45%), 295 (8.44%), 191 (5.46%), and 53 (1.52%) expanded genes were involved in dispersed, segmental, proximal, and tandem duplication, respectively. The remaining 529 expanded genes were identified as non-duplicated singletons (**Supplementary Table S5**). This phenomenon demonstrated that dispersed duplication contributed significantly to gene expansion in barley. TEs are repetitive mobile sequences scattered throughout the plant genome and have potential impact on the coding region (Schnable et al., 2009; Santana et al., 2012). Our results showed that 1,369 expanded genes were overlaped with 2,338 TEs, suggesting that these genes might be attributed to TE-mediated duplication (**Supplementary Table S6**).

### Distributions of and correlations among Ka, Ks, and Ka/Ks values in barley

**Figure 3:**
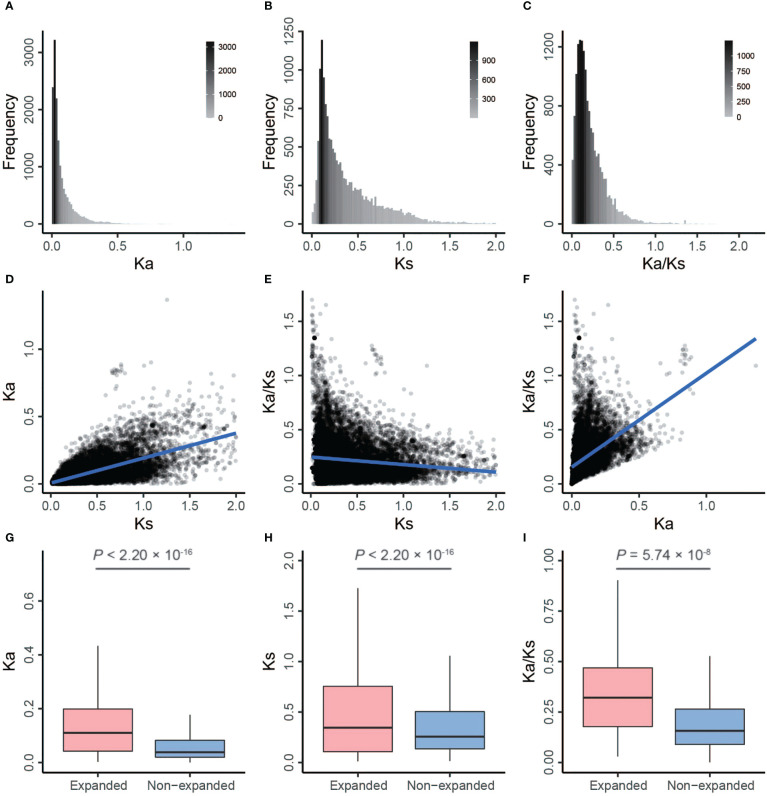
Distributions and correlation analysis of Ka, Ks, and Ka/Ks by comparing barley with rye. **(A–C)** The frequency distribution of Ka, Ks, and Ka/Ks, respectively. **(D)** The correlation between Ks (x-axis) and Ka. **(E)** The correlation between Ks (x-axis) and Ka/Ks. **(F)** The correlation between Ka (x-axis) and Ka/Ks. **(G–I)** The box plots of Ka, Ks, and Ka/Ks between expanded and non-expanded genes, respectively. The line in the box is the median value, and the lines at the bottom and top of each box are the first (lower) and third (higher) quartiles.

To provide more comprehensive insights into the selection pressures on and the evolutionary fate of barley genes, we calculated Ka, Ks, and Ka/Ks values in rye and *B. distachyon* backgrounds. The Ka/Ks ratio in the rye background ranged from 0.0010 to 2.1117 with an average of 0.2237, and the average Ka and Ks values were 0.0769 (range, 0–1.3675) and 0.3825 (range, 0.0116–1.9887), respectively (**Figures 3A–C** and **Supplementary Table S7**). Similar results were observed between barley and *B. distachyon* **(Supplementary Figures S1A–C** and **Supplementary Table S7**).

**Figure 4:**
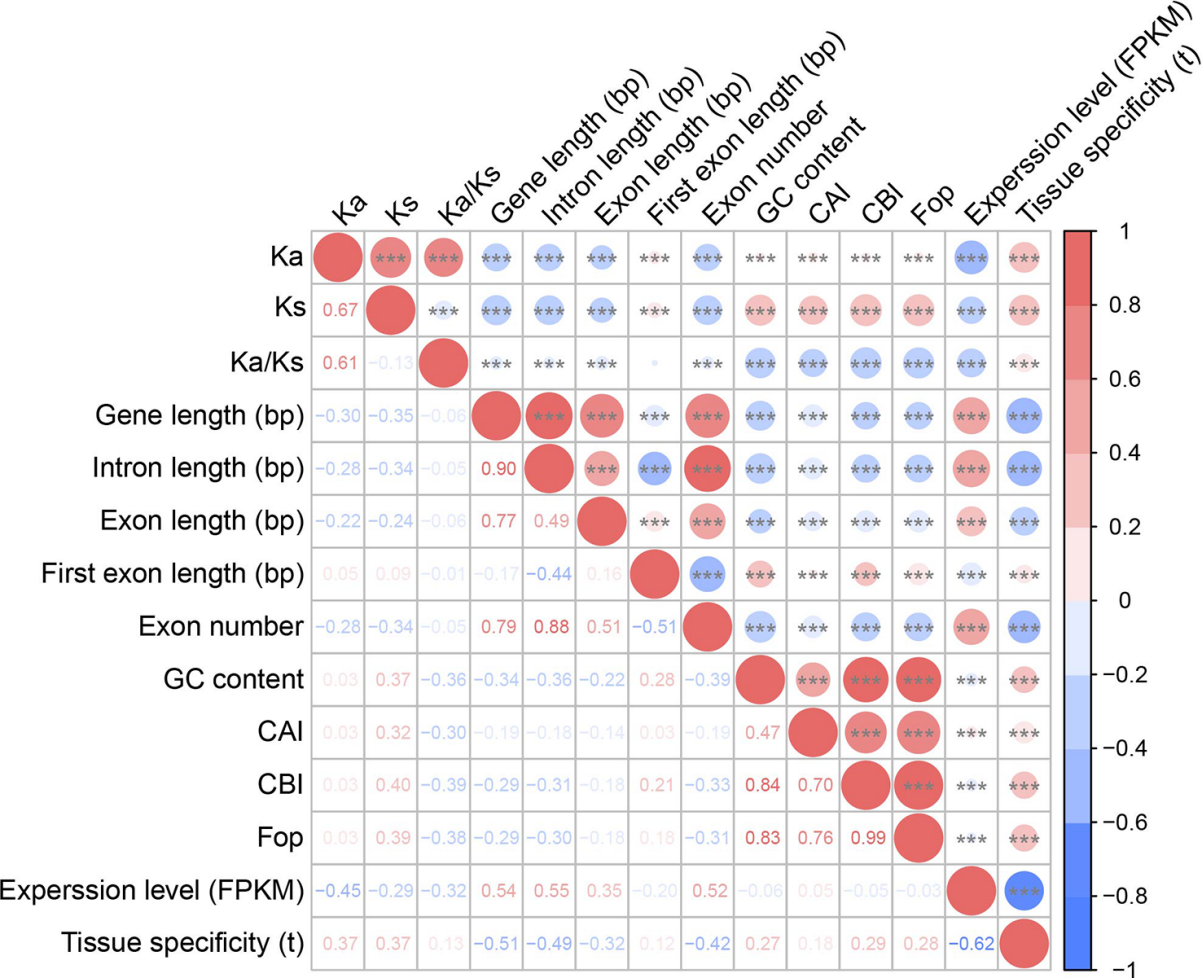
Correlations among substitution rates, gene features, codon usage bias, and expression patterns. The Ka, Ks, and Ka/Ks values were calculated between barley and rye. Upper right: the size of the circle represents the magnitude of the correlation coefficient, red indicates positive correlation, and blue indicates negative correlation. Three asterisks (***) indicate 0.001 significant difference level. Bottom left: correlation coefficients are presented as two-sided Spearman’s rank correlation test ρ.

We performed two-sided Spearman’s rank correlation tests to detect correlations between these substitution rates. Ka and Ks values were positively correlated (barley *vs*. rye: ρ = 0.67, *p* < 2.20 × 10^–16^; barley *vs*. *B. distachyon*: ρ = 0.53, *p* < 2.20 × 10^–16^; **Figures 3D**, **4**; **Supplementary Figures S1D**, **S2**; **Supplementary Tables S8**, **S9**), which was consistent with previous studies of *Pyrus* (ρ = 0.75) (Cao et al., 2019), soybean (ρ = 0.22) (Du et al., 2012), and *A. thaliana* (ρ = 0.21) (Yang and Gaut, 2011). Additionally, the Ka/Ks values were positively correlated with Ka values and negatively correlated with Ks values (**Figures 3E, F**, **4**; **Supplementary Figures S1E, F**, **S2**; **Supplementary Tables S8** and **S9**).

### Selective pressure on expanded and non-expanded genes

In general, Ka/Ks > 1, Ka/Ks < 1, and Ka/Ks = 1 indicate that proteins have experienced positive, negative (also known as purifying), and neutral selection (Hurst, 2002). Positive and negative selections are denoted as the fixation of adaptive mutations and loss of deleterious mutations, respectively (Echave et al., 2016). The average values of Ka and Ks were significantly higher for expanded genes than for non-expanded genes, but Ka/Ks was significantly higher for expanded genes than for non-expanded genes (one-sided Mann–Whitney U test, *p* < 0.01; **Figures 3G–I**; **Supplementary Figures S1G–I** and **Supplementary Table S7**). This suggests that expanded genes have evolved more rapidly and have experienced lower negative selection. In addition, a total of 102 (2.92%) positively selected and 1,917 (54.83%) negatively selected expanded genes were identified (**Supplementary Table S10**).

To determine whether gene family size affects the divergence of evolutionary rates between expanded and non-expanded genes, we classified the orthologous genes into four groups: single copy, 2–4 copies, 5–19 copies, and more than 20 copies. For single-copy orthologs, evolutionary rates of non-expanded genes were lower, and they have experienced stronger selection, indicating that they are functionally conserved (**Supplementary Figures S3**, **S4** and **Supplementary Table S11**). For multiple-copy orthologs (e.g., 2–4 and 5–19 copies), the average Ka and Ka/Ks of expanded genes were higher than those of non-expanded genes, and the opposite pattern was observed for Ks values (**Supplementary Figures S3**, **S4** and **Supplementary Table S11**). These findings demonstrate that the strength of selection on expanded genes decreases with gene family size.

**Figure 5:**
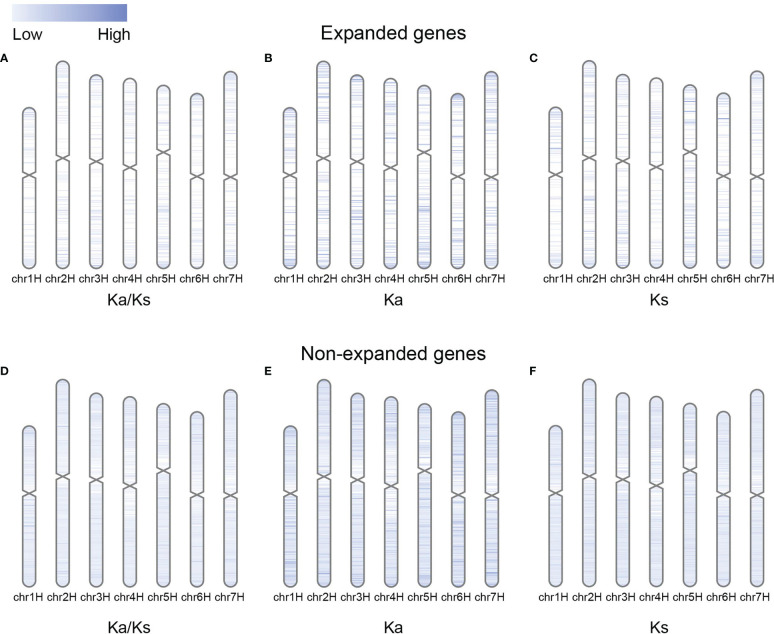
Distributions of Ka/Ks, Ka, and Ks values for expanded and non-expanded genes alongside the chromosome by comparing barley with rye. **(A–C)** Ka/Ks, Ka, and Ks values for expanded genes. **(D–F)** Ka/Ks, Ka, and Ks values for non-expanded genes.

The Ka, Ks, and Ka/Ks values of the expanded genes on the distal chromosome arm were higher than those of the expanded genes in the pericentromeric region (**Figures 5A–C**; **Supplementary Figures S5A–C**). The distribution of substitution rates and selection pressures on chromosomes was relatively undifferentiated for non-expanded genes (**Figures 5D–F**; **Supplementary Figures S5D–F**). One possible explanation is that gene duplications tended to occur on the distal chromosomal arms, and the recombination rate increased from the centromeric region to the chromosomal arm, which induced mutations in expanded genes (Gaut et al., 2007; Du et al., 2012; International Barley Genome Sequencing et al., 2012).

### Gene structure divergence of expanded and non-expanded genes

**Figure 6:**
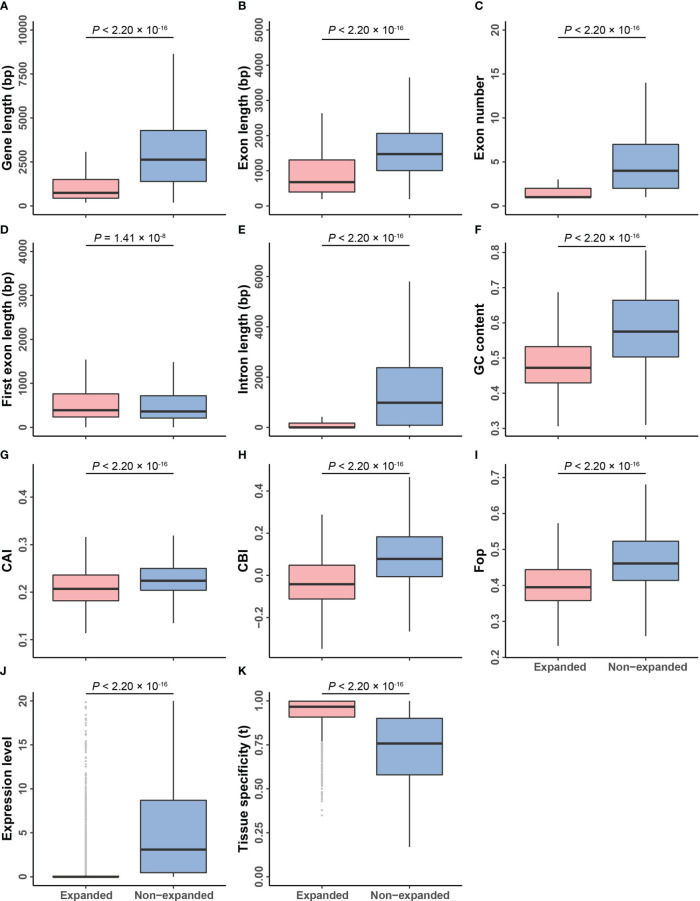
Comparisons of genomic features between expanded and non-expanded genes. **(A–K)** The box plots of gene length, exon length, exon number, first exon length, intron length, GC content, CAI, CBI, Fop, expression level, and tissue specificity between expanded and non-expanded genes. The line in the box is the median value, and the lines at the bottom and top of each box are the first (lower) and third (higher) quartiles.

To investigate how selection shapes gene structure, we compared the features of expanded and non-expanded genes. Expanded genes, including their exons and introns, were significantly shorter than non-expanded genes; however, their first exons were longer (genes: 1,415.26 *vs*. 4,229.32 bp; exons: 939.75 *vs*. 1662.06 bp; introns: 414.83 *vs*. 2567.26 bp; and first exons: 636.09 *vs*. 581.79 bp). Expanded genes also had fewer exons than non-expanded genes, and their GC content was lower (1.89 *vs*. 5.51 and 0.4805 *vs*. 0.5792, respectively) (one-sided Mann–Whitney U test, *p*-value < 0.001; **Figures 6A–F** and **Supplementary Table S12**). Single-copy genes had the longest genes, exons, and introns, suggesting that the size of the genes progressively shortened as the copy number of family members increased (**Supplementary Figures S6A–C** and **Supplementary Table S11**).

Next, we performed a correlation analysis between evolutionary rate and gene properties. Both Ka and Ks values were significantly negatively correlated with gene and exon and intron lengths but positively correlated with the first exon length and GC content, indicating that the evolutionary rate might affect the structure of genes in barley (two-sided Spearman’s rank correlation test, *p*-value < 0.05, **Figure 4**; **Supplementary Figure S2**; **Supplementary Tables S8**, **S9**). However, there was no strong correlation between Ka/Ks values and gene properties.

### Expression levels and tissue specificity of expanded and non-expanded genes

The expression levels of expanded genes were lower than those of non-expanded genes (2.19 *vs*. 17.20; one-sided Mann–Whitney U test, *p*-value < 2.20 × 10^–16^; **Figure 6J** and **Supplementary Table S12**). Expression pattern analysis showed that 0.74% of the expanded genes were highly expressed (FPKM ≥ 50), but 92.51% of them were weakly expressed (FPKM ≤ 3) (**Supplementary Table S13**). In contrast, the expression of 6.52% of non-expanded genes was high, and the expression of 39.33% of these genes was low. These results suggest that the expression patterns of expanded and non-expanded genes have diverged (one-sided Fisher’s exact test, *p*-value < 2.20 × 10^–16^).

We analyzed whether the expanded genes showed tissue-specific expression. Categorical and overall tissue-specific genes comprised 9.55% and 90.45% of the expanded genes, respectively (**Supplementary Table S13**). Categorical tissue-specific genes and overall tissue-specific genes comprised 3.43% and 96.57% of non-expanded genes, respectively. The tissue specificity (τ) of expanded genes was significantly higher than that of non-expanded genes (one-sided Fisher’s exact test, *p*-value < 2.20 × 10^–16^, **Figure 6K** and **Supplementary Table S13**). Similar to previous studies, we found that gene expression levels decreased as the copy number and tissue specificity of expression increased (**Supplementary Figures S6J, K** and **Supplementary Table S11**) (De La Torre et al., 2015).

Furthermore, we examined the correlation between selection pressures and gene expression patterns. There was a significant negative correlation between the Ka/Ks ratio and expression levels (two-sided Spearman’s rank correlation test, ρ = –0.32, *p*-value < 2.20 × 10^–16^; **Figure 4** and **Supplementary Table S8**). The Ka/Ks ratio was positively correlated with tissue specificity (two-sided Spearman’s rank correlation test, ρ = 0.13, *p*-value < 2.20 × 10^–16^; **Figure 4** and **Supplementary Table S8**).

### Comparison of codon usage bias between expanded and non-expanded genes

Due to the degeneracy of the genetic code, most of the amino acids are coded by several synonymous codons. The preferential usage of codons is a phenomenon in which synonymous codons are used more frequently than others, and the usage preference in plant genomes has been shaped by natural selection to mediate adaptation to the environment (Morton, 2003; Behura and Severson, 2013; Li Y. et al., 2021). The CAI, CBI, and Fop for each gene were calculated to determine whether selection pressure, gene structure, and expression patterns affect codon usage bias. The average CAI, CBI, and Fop were all significantly higher for non-expanded genes than for expanded genes (one-sided Mann–Whitney U test, *p*-value < 2.20 × 10^–16^; **Figures 6G–I** and **Supplementary Table S12**). As copy number increased, these three codon bias indicators for expanded genes gradually decreased; similar results have been obtained in yeast, where codon usage bias was lower for more rapidly evolving genes (**Supplementary Figures S6G–I** and **Supplementary Table S11**) (Bu et al., 2011).

Correlation analysis revealed that these three indicators were significantly positively correlated with Ks rather than Ka and negatively correlated with Ka/Ks (one-sided Mann–Whitney U test, *p*-value < 2.20 × 10^–16^; **Figure 4**; **Supplementary Figure S2** and **Supplementary Tables S8**, **S9**). Additionally, these indicators were negatively correlated with gene length, exon length, number of exons, and intron length but positively correlated with first exon length and the tissue specificity of expression (one-sided Mann–Whitney U test, *p*-value < 0.001; **Figure 4** and **Supplementary Table S8**).

### Population structure and genetic diversity of expanded and non-expanded genes

**Figure 7:**
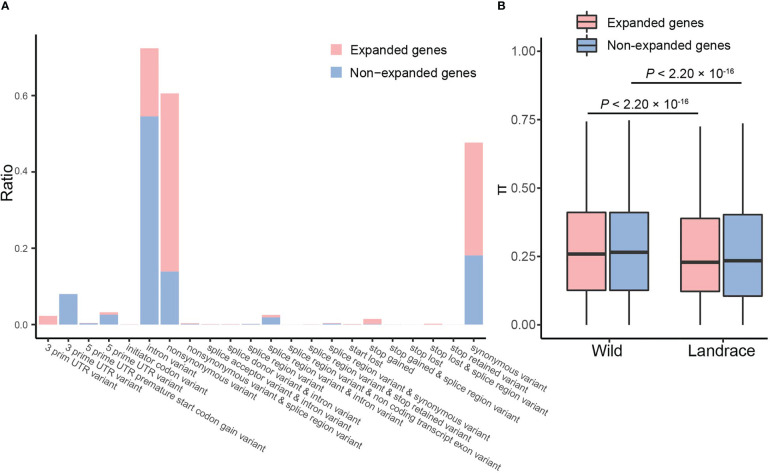
Nucleotide diversity analysis for expanded and non-expanded genes. **(A)** Frequency distributions of various variants. Pink and blue columns represent expanded and non-expanded genes, respectively. **(B)** The box plots of nucleotide diversity.

To characterize the landscape of genetic variation, a total of 215 publicly available resequenced samples of barley were used. We obtained 14,959 and 572,331 SNPs in expanded and non-expanded genes, respectively (**Figure 7A** and **Supplementary Table S14**). For expanded genes, the most prominent variants were non-synonymous variants (46.67%), synonymous variants (29.61%), and intron variants (17.86%). In contrast, the dominant variant in non-expanded genes was intron variants (54.52%), followed by synonymous variants (18.08%) and non-synonymous variants (13.90%). These results suggest that a large number of non-synonymous mutants have been retained in expanded genes and that these loci were likely associated with the adaptive evolution of the barley genome.

**Figure 8:**
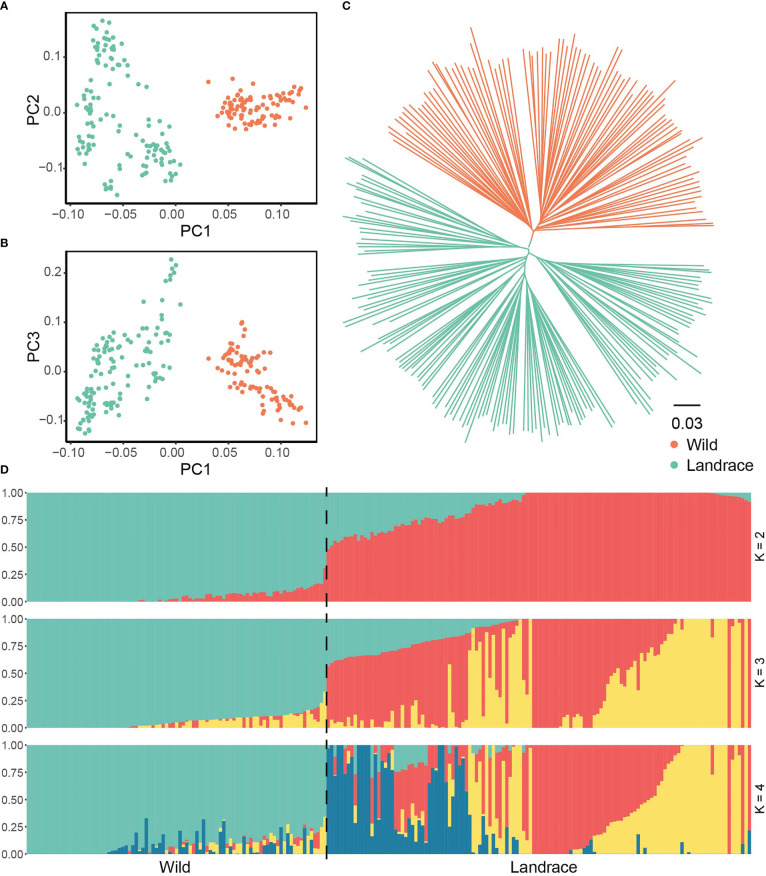
Population structure between wild barley and landrace based on SNPs in expanded genes. **(A)** Principal component analysis PC1 *vs*. PC2. **(B)** Principal component analysis PC1 *vs*. PC3. **(C)** The NJ phylogenetic tree. **(D)** Population structure with K ranging from 2 to 4.

We further elucidated the evolutionary trajectory of expanded and non-expanded genes during barley domestication. In the PCA, the first principal component, which was predominantly correlated with the divergence between wild and landrace barley, explained 6.66% of the total genetic variance in expanded genes. The geographical origins were correlated with the second and third eigenvectors, which explained 3.11% and 2.76% of the variance, respectively (**Figures 8A, B** and **Supplementary Table S15**). The phylogenetic tree further supported the observations of the PCA and provided more robust insights into the relationships among accessions (**Figure 8C**). When the putative number of populations was set to 2, divergence was observed between wild and landrace barley (**Figure 8D**). No significant difference was observed in population structure according to non-expanded genes (**Supplementary Figure S7** and **Supplementary Table S15**).

Estimates of genetic diversity are important for evolutionary and genetic research in plants (Mao et al., 2019). Nucleotide diversity of expanded and non-expanded genes significantly decreased (~5.46%) from wild barley (0.2636) to landrace barley (0.2492) (**Figure 7B** and **Supplementary Table S16**). Specifically, decreases in nucleotide diversity were significantly more pronounced in expanded genes than non-expanded genes, which suggests that expanded genes have undergone more severe genetic bottlenecks than non-expanded genes during barley domestication (~6.00% *vs*. ~5.4%; **Figure 7B** and **Supplementary Table S16**).

### Functional enrichment analysis of expanded and non-expanded genes

To reveal the potential biological functions of expanded and non-expanded genes, we performed GO, KEGG, and PO enrichment analyses. For expanded genes, a total of 185, 49, and 84 GO terms were significantly enriched in the biological process (BP), cellular component (CC), and molecular function (MF) categories, respectively (**Supplementary Table S17**). A large number of terms were enriched in response to stimulus (GO:0050896), signal transduction (GO:0007165), defense response (GO:0006952), catalytic activity (GO:0003824), and kinase activity (GO:0016301) (**Supplementary Figure S8**). KEGG analysis revealed that expanded genes were enriched in oxidative phosphorylation, photosynthesis, and DNA repair (**Supplementary Figure S9** and **Supplementary Table S18**). PO enrichment analysis revealed that expanded genes were enriched in mesophyll cell (PO:0004006), leaf (PO:0025034), lateral root (PO:0020121), and embryo sac central cell (PO:0020090) (**Supplementary Figure S10** and **Supplementary Table S19**). For non-expanded genes, a total of 194, 79, and 51 GO terms were enriched in BP, CC, and MF, such as primary metabolic process (GO:0044238), regulation of biological process (GO:0050789), binding (GO:0005488), and transcription regulator activity (GO:0140110) (**Supplementary Figure S11** and **Supplementary Table S20**). Moreover, non-expanded genes were highly enriched in translation, transcription, and various metabolism KEGG pathways (**Supplementary Figure S12** and **Supplementary Table S21**). PO enrichment analysis showed that non-expanded genes were significantly enriched in pollen tube cells (PO:0025195), plant sperm cells (PO:0000084), and seed germination stage (PO:0007057) (**Supplementary Figure S13** and **Supplementary Table S22**). These results indicate that these non-expanded genes were involved in basic metabolic and reproductive development processes and that expanded genes play key roles in the response to stresses.

### Differentially expressed genes and co-expression network of candidate expanded genes

Expression profiles provide insights into the potential functions of genes in plant species (Yang et al., 2022). The expression of the expanded gene *HORVU.MOREX.r3.1HG0003780* was upregulated under drought treatment (**Supplementary Figure S14B** and **Supplementary Table S24**). The expression of four expanded genes in OG0000152 (*HORVU.MOREX.r3.7HG0738450*, *HORVU.MOREX.r3.7HG0738520*, *HORVU.MOREX.r3.7HG0738470*, and *HORVU.MOREX.r3.7HG0738490*) was upregulated in the roots under water-deficit conditions (**Supplementary Figure S14** and **Supplementary Table S24**). The expression of *HORVU.MOREX.r3.7HG0738470* and *HORVU.MOREX.r3.7HG0738490* was induced by salt treatment. These results demonstrate that the functionally divergent OG0000152 family genes might play essential roles in the response to adverse stresses.

**Figure 9:**
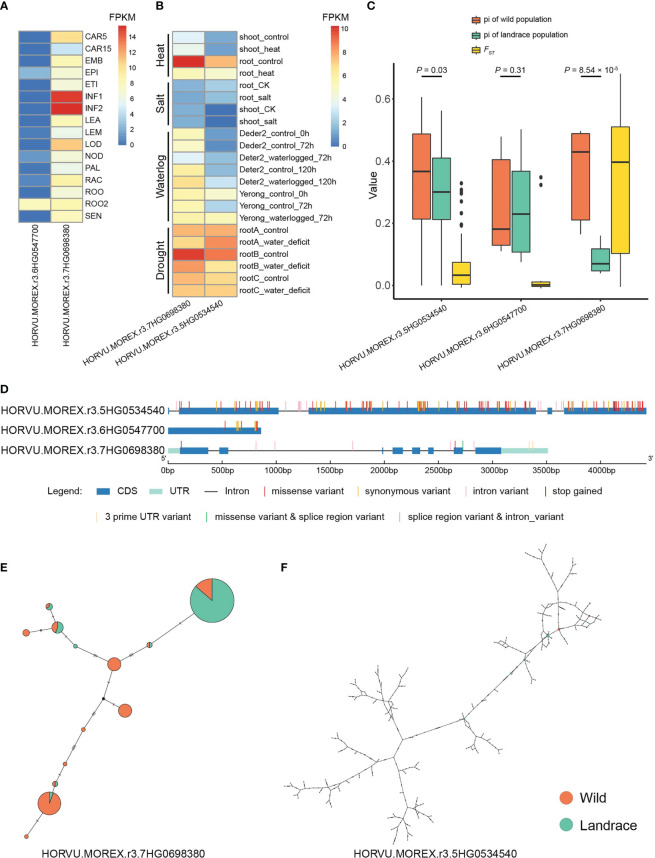
Expression pattern and median-joining haplotype networks of TF-type expanded genes. **(A)** Expression pattern of candidate genes in different tissues/stages. CAR15, bracts removed grains at 15DPA; CAR5, bracts removed grains at 5DPA; EMB, embryos dissected from 4-day-old germinating grains; EPI, epidermis with 4 weeks old; ETI, etiolated from 10-day-old seedling; INF1, young inflorescences with 5 mm; INF2, young inflorescences with 1–1.5 cm; LEA, shoot with the size of 10 cm from the seedlings; LEM, lemma with 6 weeks after anthesis; LOD, lodicule with 6 weeks after anthesis; NOD, developing tillers at six-leaf stage; PAL, inflorescences, palea (6 weeks after pollination); RAC, inflorescences, rachis (5 weeks after pollination); ROO, roots from the seedlings with 17 and 28 days old after planting; ROO2, roots (4 weeks after pollination); SEN, senescing leaf. **(B)** Expression pattern of candidate genes under different stresses. **(C)** The boxplots of pi and FST values for candidate genes. **(D)** Distribution of nucleotide variants within the candidate genes. **(E, F)** Median-Joining haplotype networks of candidate genes in wild barley and landrace populations. The circle size represents the number of accessions holding a particular haplotype. The orange and green circles refer to wild barley and landrace accessions, respectively.

**Figure 10:**
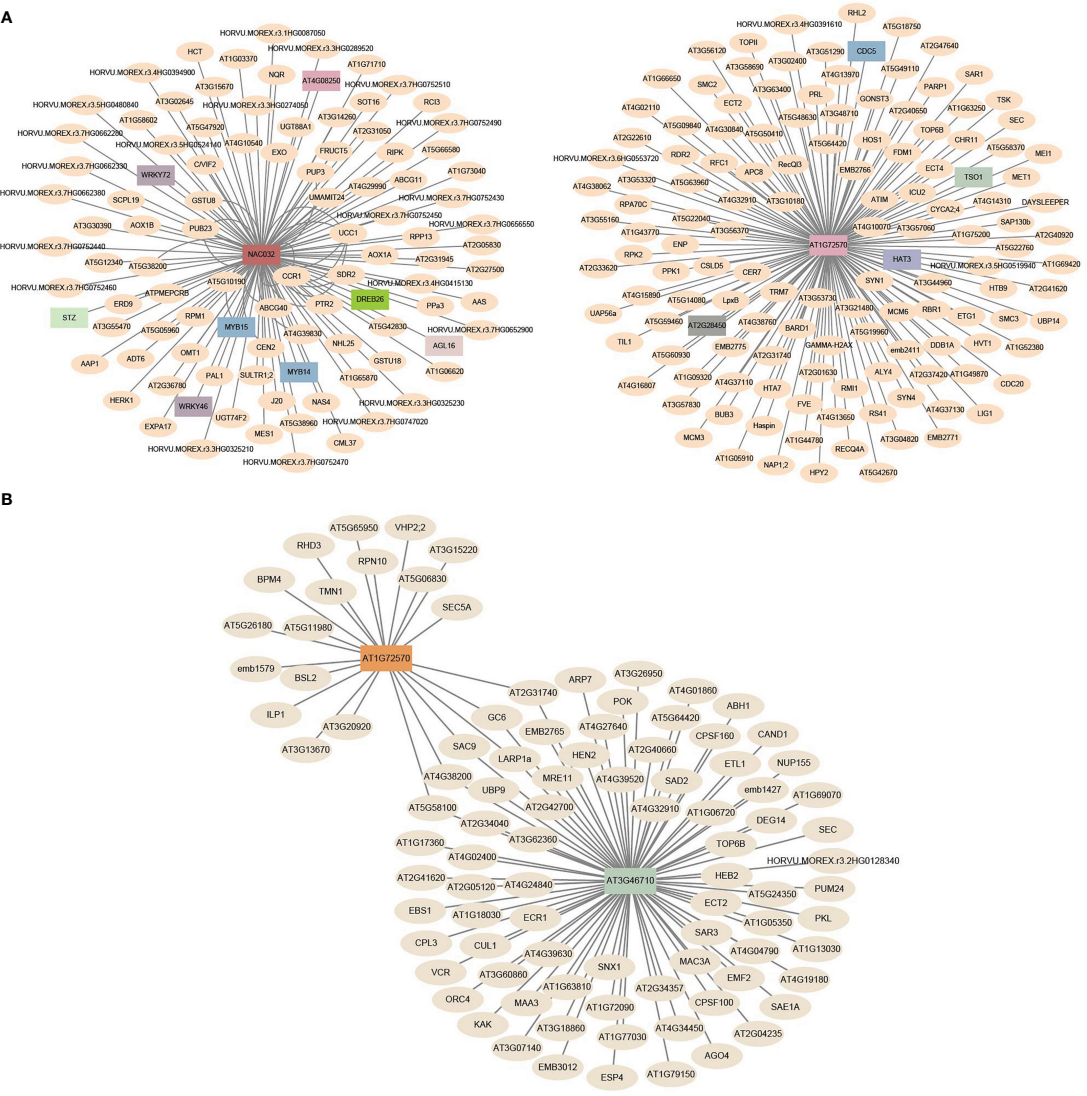
The co-expression network of expanded genes with other TF-type genes. **(A)** Co-expression network in different tissues/stages. **(B)** Co-expression network under different stresses. The barley genes were annotated with homologs of *A. thaliana*.

TFs activate or repress the expression of target genes by binding to specific DNA sequences during various biological processes (Ibarra et al., 2020). In this study, a total of 83 expanded genes (11 orthogroups) were identified as TFs (**Supplementary Table S25**). To explore their potential biological functions and regulatory networks, we constructed co-expression networks with TFs as central nodes. According to pairwise correlations of gene expression across samples, co-expression networks were constructed to characterize regulatory changes in gene expression (Langfelder and Horvath, 2008). WGCNA revealed 18 TF-type expanded genes that were central hubs in the co-expression networks and we further explored their expression profiles, variant sites, and haplotypes. (**Figures 9A–F** and **Supplementary Table S25**). Two central hub TFs, *HORVU.MOREX.r3.7HG0698380* (homologous to *AT1G72570*, encoded AP2 TF) and *HORVU.MOREX.r3.6HG0547700* (homologous to *NAC032*, encoded NAC TF), were co-expressed with 135 and 118 genes, respectively, which formed a network composed of 253 connections (**Figure 10A** and **Supplementary Table S26**). Another co-expression network with *HORVU.MOREX.r3.7HG0698380* (homologous to *AT1G72570*, encoded AP2 TF) and *HORVU.MOREX.r3.6HG0547700* (homologous to *NAC032*, encoded NAC TF) as central nodes contained 100 connections, and these two central genes were linked with 78 and 22 genes, respectively (**Figure 10B** and **Supplementary Table S27**).

The expression patterns for these hub genes were further explored using data from 16 different development tissues/stages and under four types of stress (**Figures 9A, B** and **Supplementary Figures S15**-**S17**). The expression patterns of these genes displayed high tissue specificity, with an average τ-value of 0.7675, ranging from 0.4295 to 0.9907 (**Supplementary Table S25**). *HORVU.MOREX.r3.7HG0698380* was preferentially expressed in inflorescences and was differentially expressed under heat, waterlogging, and drought stress (**Figures 9A, B** and **Supplementary Table S25**). In addition, *HORVU.MOREX.r3.6HG0547700* was primarily expressed in the roots, and the expression of *HORVU.MOREX.r3.5HG0534540* was induced by heat, waterlogging, and drought stress.

The nucleotide diversity of *HORVU.MOREX.r3.7HG0698380* and *HORVU.MOREX.r3.5HG0534540* was significantly higher in wild barley than in landrace accessions (one-sided Mann–Whitney U test, *p*-value < 0.05) (**Figure 9C** and **Supplementary Table S25**). Specially, a total of 133, 12, and 8 SNPs were identified in *HORVU.MOREX.r3.5HG0534540*, *HORVU.MOREX.r3.6HG0547700*, and *HORVU.MOREX.r3.7HG0698380*, respectively (**Figure 9D** and **Supplementary Table S25**). A relatively high *F_ST_* index (0.3454) between wild and landrace accessions was observed in *HORVU.MOREX.r3.7HG0698380*, indicating differentiation during domestication (**Figure 9C** and **Supplementary Table S25**). *HORVU.MOREX.r3.7HG0698380* possessed 13 haplotypes, six of which were specific to the wild population (**Figure 9E** and **Supplementary Table S25**). By contrast, *HORVU.MOREX.r3.5HG0534540* possessed 162 haplotypes (**Figure 9F** and **Supplementary Table S25**).

## Discussion

### Expanded genes evolved more rapidly and experienced lower negative selection

The selection pressure on expanded genes determines their evolutionary fates after duplication events (Jacquemin et al., 2014). However, the roles of selection in shaping the evolutionary history of barley-expanded genes remain unclear. In this study, we obtained 27,438 orthogroups from the protein-coding genes of seven Poaceae species. A total of 3,496 expanded and 21,537 non-expanded genes were identified. To estimate their evolutionary rates, we calculated the Ka, Ks, and Ka/Ks values for each homologous gene pair. The average values of Ka and Ks for expanded genes were greater than those for non-expanded genes, which indicated that expanded genes evolved much faster. These results were similar to those of previous studies showing that duplicated genes had higher evolutionary rates than single-copy genes (Pegueroles et al., 2013; O'Toole et al., 2018; Vance et al., 2022).

A reliable model has been proposed to explain the evolutionary fate of duplicated genes. In this model, gene copies are retained under negative selection due to short-term gene dosage advantage in the early phase after duplication; in the later stage of duplication, a few copies experience positive selection and acquire new functions (Lynch and Conery, 2000; Pegueroles et al., 2013; Gout and Lynch, 2015). Our analysis revealed that the average Ka/Ks value of expanded genes was approximately twice as large as that of non-expanded genes, and this ratio gradually increased as the copy number increased. These observations suggest that there were numerous copies of expanded genes under relaxed negative selection, but non-expanded genes evolved more slowly than multi-copy expanded genes owing to functional constraints and stronger negative selection. We also found that a large proportion of expanded genes experienced negative selection rather than positive selection. Similar findings have been made in previous studies (Lynch and Conery, 2000). Therefore, we hypothesized that most of the expanded genes have been subfunctionalized or pseudogenized in the barley genome. Expanded genes are unlikely to undergo neofunctionalization because most non-synonymous mutations are deleterious (Jacquemin et al., 2014; de Oliveira et al., 2019).

### Gene properties, codon usage bias, and expression patterns contribute to the divergent evolutionary rates of expanded genes

An increasing number of studies have shown that the evolutionary rate of proteins is affected by multiple factors, such as gene structure, codon usage bias, and expression levels (Drummond et al., 2005; Zhang and Yang, 2015; Echave et al., 2016). The potential factors shaping the evolutionary rate of expanded and non-expanded genes were further explored. Gene expression has been reported to affect both the fate of duplicated genes and evolutionary rates (Zhang and Yang, 2015; Panchy et al., 2016). In this study, we found that expanded genes, including their exons and introns, were shorter, but they had longer first exons and lower expression levels. Correlation analysis showed that Ka and Ks values were negatively correlated with gene length, intron length, exon length, and the number of exons. Several studies support these observations; for example, duplicate genes with shorter CDS lengths have also been detected in primates, suggesting that the expansion of longer genes might be more costly (O'Toole et al., 2018). The shorter first exons of non-expanded genes act as position-dependent transcriptional enhancers by activating histone modifications, including H3K4me3 and H3K9ac, to increase expression levels (Bieberstein et al., 2012; Aljohani et al., 2020).

Highly expressed proteins are usually encoded by genes with stronger codon usage bias (Yang et al., 2019). Our results revealed that the expression levels of expanded genes were lower; however, the tissue specificity of the expression of expanded genes was higher, and the codon usage bias of these genes was weaker. We also noticed that Ks, rather than Ka, was positively correlated with codon usage bias, which indicated that synonymous substitutions affect the formation of codon usage bias in barley. The Ka/Ks ratio was negatively correlated with the expression level of expanded genes but positively correlated with the tissue specificity of the expression of expanded genes. This indicates that the lower selection on expanded genes was responsible for their lower expression level and indicates that the neofunctionalization of expanded genes is rare. As gene family size increased, gene expression levels decreased, and the tissue specificity of the expression of genes increased, which can be explained by the fact that functional redundancy after gene duplication may be altered by decreases in expression levels. This suggests that single-copy genes tend to be expressed at a high level compared with multi-copy expanded genes (De Smet et al., 2013; De La Torre et al., 2015).

### Non-synonymous variants played essential roles in the reduction in the genetic diversity of expanded genes during barley domestication

Crop domestication refers to the process in which wild ancestors experience long-term artificial selection to acquire traits that facilitate harvest or increase yields in landraces and cultivars (Yu and Li, 2022). Crop domestication has resulted in the loss of genetic diversity in modern cultivars compared with their wild ancestors, a phenomenon that is often referred to as the “domestication bottleneck” (Zhang et al., 2021). The nucleotide diversity of landrace accessions was decreased by 27% across the whole genome compared with wild barley (Russell et al., 2016). However, little is known regarding changes in expanded and non-expanded genes during barley domestication.

Analysis of 587,290 SNPs revealed a significant decrease in average nucleotide diversity of ~6.00% and ~5.4% for expanded and non-expanded genes, respectively, from wild accessions to landraces. The non-synonymous variant (46.67%) accounted for most of the expanded genes, but the intron variant was the most common variant type among non-expanded genes. We thus inferred that the divergent variant types might have led to differences in genetic diversity and evolutionary rates in expanded and non-expanded genes. Because non-expanded genes tend to be housekeeping genes, multiple non-synonymous variants might be detrimental to the survival of barley. However, beneficial non-synonymous variants might have been retained in several expanded genes during barley domestication because they enhance adaptation to the environment or confer excellent agronomic traits.

### Expanded genes might play essential roles in responses to abiotic and biotic stress

WGCNA is a systems biology method for the identification of association patterns, functional modules, and hub genes (Langfelder and Horvath, 2008). TFs are the key regulators involved in transcriptional regulation (Ibarra et al., 2020). Our analysis yielded co-expression networks with TF-type expanded genes as the central nodes. The hub genes *HORVU.MOREX.r3.6HG0547700*, *HORVU.MOREX.r3.7HG0698380*, and *HORVU.MOREX.r3.5HG0534540* encoded NAC, AP2, and B3 TFs, respectively. The homologous gene of *HORVU.MOREX.r3.6HG0547700* in *A. thaliana* was *ANAC032*, which regulates root growth in response to reactive oxygen species signaling (Maki et al., 2019). In rice, overexpression of its homolog *OsNAC9* altered root architecture and increased drought resistance and grain yield (Redillas et al., 2012). *HORVU.MOREX.r3.7HG0698380* was highly expressed in inflorescences and was differentially expressed under different types of stress (**Supplementary Figure S17** and **Supplementary Table S25**). The expression pattern of its homologous gene *AIL1* in *A. thaliana* was similar (Nole-Wilson et al., 2005). Furthermore, the differentiation of haplotypes under strong selection pressure suggests that these candidates have potentially played an important role in the domestication process in barley (**Figure 9C** and **Supplementary Table S25**).

B3 superfamily genes contain at least one conserved B3 DNA-binding domain that interacts directly with *cis*-acting elements, and they are involved in plant growth, hormone signaling, and the response to biotic/abiotic stresses (Swaminathan et al., 2008; Fu et al., 2014; Chen et al., 2015). The B3 gene family has undergone a significant expansion, and this has been mainly mediated by tandem duplication. These genes were clustered into the orthogroups OG0001472 (only containing *HORVU.MOREX.r3.5HG0534540*) and OG0000195 (containing 41 genes) (**Supplementary Figure S18** and **Supplementary Table S24**). Expression analysis revealed that the genes in OG0000195 might have undergone pseudogenization, as they were not highly expressed in any tissue/stage or under any stress; by contrast, *HORVU.MOREX.r3.5HG0534540* in OG0001472 was highly expressed under heat stress. In *A. thaliana*, the homologous gene of *HORVU.MOREX.r3.5HG0534540* shows sequence and functional diversity in pathogen recognition (Rose et al., 2004), suggesting that neofunctionalization or subfunctionalization has driven the origin of this gene.

Other candidate expanded genes were obtained aside from TF-type expanded genes. Plants have evolved disease resistance (*R*) genes to specifically recognize and confer resistance to pathogens and insects (Zhang et al., 2016; Kourelis and van der Hoorn, 2018). *R* genes can be classified according to their protein domains and structures. The vast majority of *R* genes are *NBS–LRRs* (Marone et al., 2013). A total of 44 out of 411 *NBS-LRR*s were associated with expansion in the barley genome (**Supplementary Table S28**). The expanded *NBS-LRR*s were clustered into seven orthogroups and mainly located on chromosomes 7 and 1 through tandem duplications (**Supplementary Figure S19**). Among the expanded *NBS-LRR* genes, *HORVU.MOREX.r3.5HG0495560* has undergone a domestication bottleneck, including a severe loss of genetic diversity (**Supplementary Table S25**).

In addition, five expanded genes were found to be differentially expressed under various types of stress, suggesting that they could be used for the molecular breeding of plants with enhanced stress resistance (**Supplementary Figure S14B** and **Supplementary Table S24**). The expression of another hub gene, *HORVU.MOREX.r3.3HG0272100*, was upregulated in response to heat and drought (**Supplementary Figure S17B** and **Supplementary Table S25**). A significant reduction in nucleotide diversity was observed in *HORVU.MOREX.r3.3HG0272100*. These domestication-related genes provide valuable genetic resources that could be used to enhance the agronomic traits of crops.

## Data availability statement

The original contributions presented in the study are included in the article/**Supplementary Material**. Further inquiries can be directed to the corresponding author.

## Author contributions

WT: formal analysis, visualization. RL: formal analysis. TL: methodology. ZL: supervision. YL: investigation, writing—original draft. LC: data curation, writing—original draft, supervision. All authors contributed to the article and approved the submitted version.

## Supplementary material

Supplementary Figure 1 Distributions and correlation analysis of Ka, Ks, and Ka/Ks by comparing barley with *B. distachyon*. **(A-C)** The frequency distribution of Ka, Ks, and Ka/Ks, respectively. **(D)** The correlation between Ks (x-axis) and Ka. **(E)** The correlation between Ks (x-axis) and Ka/Ks. **(F)** The correlation between Ka (x-axis) and Ka/Ks. **(G-I)** The box plots of Ka, Ks, and Ka/Ks between expanded and non-expanded genes, respectively. The line in the box is the median value, and the lines at the bottom and top of each box are the first (lower) and third (higher) quartiles.

Supplementary Figure 2 Correlations among substitution rates, gene features, codon usage bias, and expression patterns. The Ka, Ks, and Ka/Ks values were calculated between barley and *B. distachyon*. Upper Right: the size of the circle represents the magnitude of the correlation coefficient, red indicates positive correlation, and blue indicates negative correlation. Three asterisks (***) indicate 0.001 significant difference level. Bottom Left: correlation coefficients are presented as two-sided Spearman’s rank correlation test ρ.

Supplementary Figure 3 Group boxplots of Ka **(A)**, Ks **(B)**, and Ka/Ks **(C)** by comparing barley with rye. The line in the box is the median value, and the lines at the bottom and top of each box are the first (lower) and third (higher) quartiles. Multiple comparison was performed by LSD test.

Supplementary Figure 4 Group boxplots of Ka **(A)**, Ks **(B)**, and Ka/Ks **(C)** by comparing barley with *B. distachyon*. The line in the box is the median value, and the lines at the bottom and top of each box are the first (lower) and third (higher) quartiles. Multiple comparison was performed by LSD test.

Supplementary Figure 5 Distributions of Ka, Ks, and Ka/Ks values for expanded and non-expanded genes alongside the chromosome by comparing barley with *B. distachyon*.

Supplementary Figure 6 Comparisons of genomic features between expanded and non-expanded genes grouped by copy number. The line in the box is the median value, and the lines at the bottom and top of each box are the first (lower) and third (higher) quartiles. Multiple comparison was performed by LSD test.

Supplementary Figure 7 Population structure between wild barley and landrace based on SNPs in non-expanded genes. **(A)** Principal component analysis PC1 *vs.* PC2. **(B)** Principal component analysis PC1 *vs.* PC3. **(C)** The NJ phylogenetic tree. **(D)** Population structure with K ranging from 2 to 4.

Supplementary Figure 8 GO enrichment analysis of expanded genes. The top 10 GO enrichment terms are displayed for each category.

Supplementary Figure 9 KEGG pathway enrichment analysis of expanded genes.

Supplementary Figure 10 PO enrichment analysis of expanded genes.

Supplementary Figure 11 GO enrichment analysis of non-expanded genes. The top 10 GO enrichment terms are displayed for each category.

Supplementary Figure 12 KEGG pathway enrichment analysis of non-expanded genes.

Supplementary Figure 13 PO enrichment analysis of non-expanded genes.

Supplementary Figure 14 Expression pattern of differentially expressed expanded genes. Detailed annotation of tissues/stages is the same as.

Supplementary Figure 15 Expression pattern of *NBS-LRR*s in different tissues/stages. Detailed annotation of tissues/stages is the same as.

Supplementary Figure 16 Expression pattern of *NBS-LRR*s under different stresses.

Supplementary Figure 17 Expression pattern of top 10 hub genes in tissue/stage **(A)** and stress **(B)** co-expression networks. Detailed annotation of tissues/stages is the same as.

Supplementary Figure 18 Distribution of the B3 genes on barley chromosomes. Chromosome numbers are shown at the top of each chromosome. The scale (Mb) notes on the left. The genes marked in red are expanded genes in barley.

Supplementary Figure 19 Distribution of *NBS-LRR*s on barley chromosomes. Chromosome numbers are shown at the top of each chromosome. The scale (Mb) notes on the left. The genes marked in red are expanded genes in barley.
