# Supplementary figures and images for: The evolutionary patterns, expression profiles, and genetic diversity of expanded genes in barley
Source: Front Plant Sci. 2023 Apr 26;14:1168124. doi: 10.3389/fpls.2023.1168124 (PMC10171312; doi:10.3389/fpls.2023.1168124)

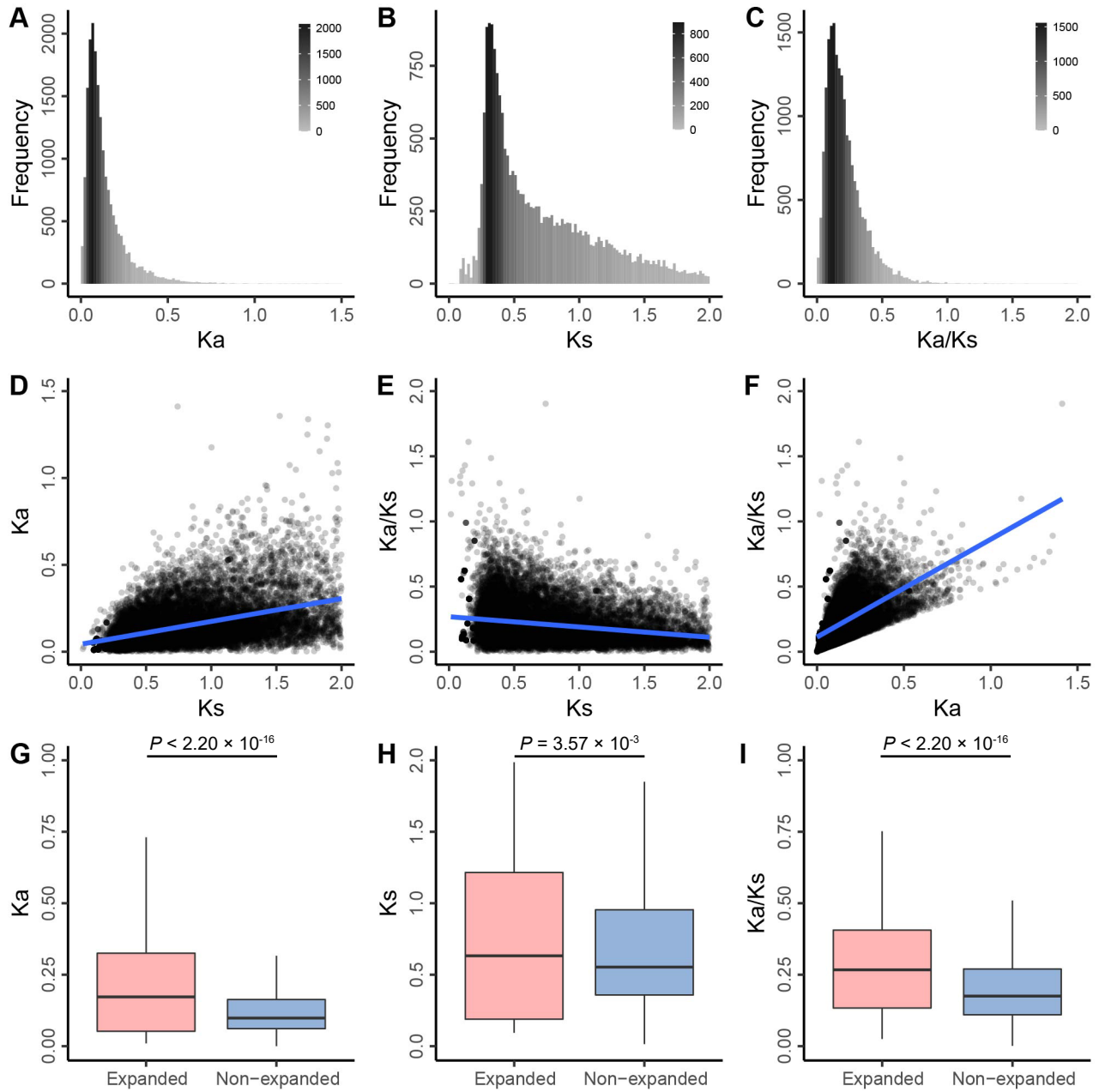

Supplement: Supplementary Figure 1 — Distributions and correlation analysis of Ka, Ks, and Ka/Ks by comparing barley with B. distachyon. (A-C) The frequency distribution of Ka, Ks, and Ka/Ks, respectively. (D) The correlation between Ks (x-axis) and Ka. (E) The correlation between Ks (x-axis) and Ka/Ks. (F) The correlation between Ka (x-axis) and Ka/Ks. (G-I) The box plots of Ka, Ks, and Ka/Ks between expanded and non-expanded genes, respectively. The line in the box is the median value, and the lines at the bottom and top of each box are the first (lower) and third (higher) quartiles. [file DataSheet_1.zip › Supplementary_Figures/Supplementary Figure 1.pdf]

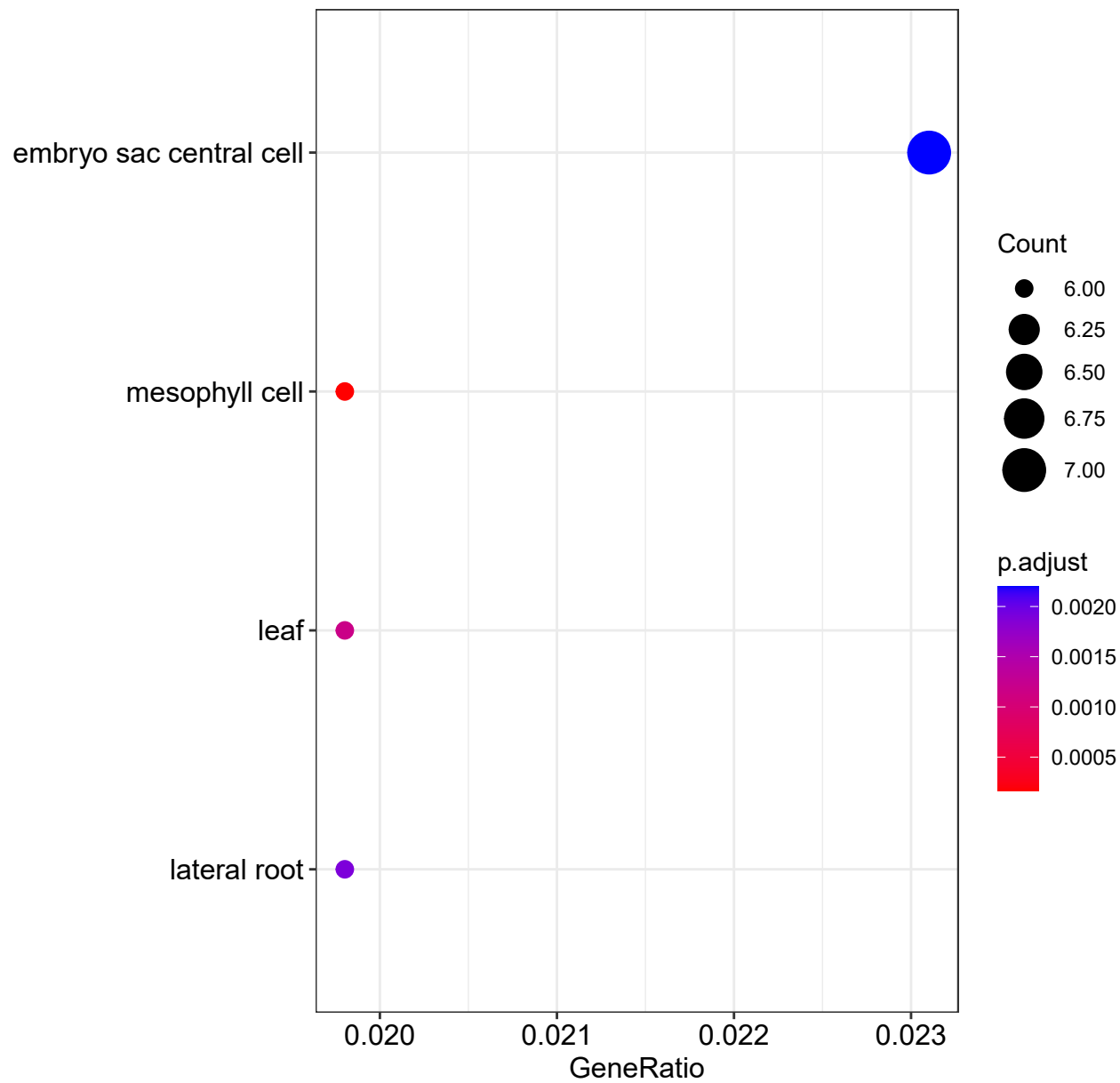

Supplement: Supplementary Figure 1 — Distributions and correlation analysis of Ka, Ks, and Ka/Ks by comparing barley with B. distachyon. (A-C) The frequency distribution of Ka, Ks, and Ka/Ks, respectively. (D) The correlation between Ks (x-axis) and Ka. (E) The correlation between Ks (x-axis) and Ka/Ks. (F) The correlation between Ka (x-axis) and Ka/Ks. (G-I) The box plots of Ka, Ks, and Ka/Ks between expanded and non-expanded genes, respectively. The line in the box is the median value, and the lines at the bottom and top of each box are the first (lower) and third (higher) quartiles. [file DataSheet_1.zip › Supplementary_Figures/Supplementary Figure 10.pdf]

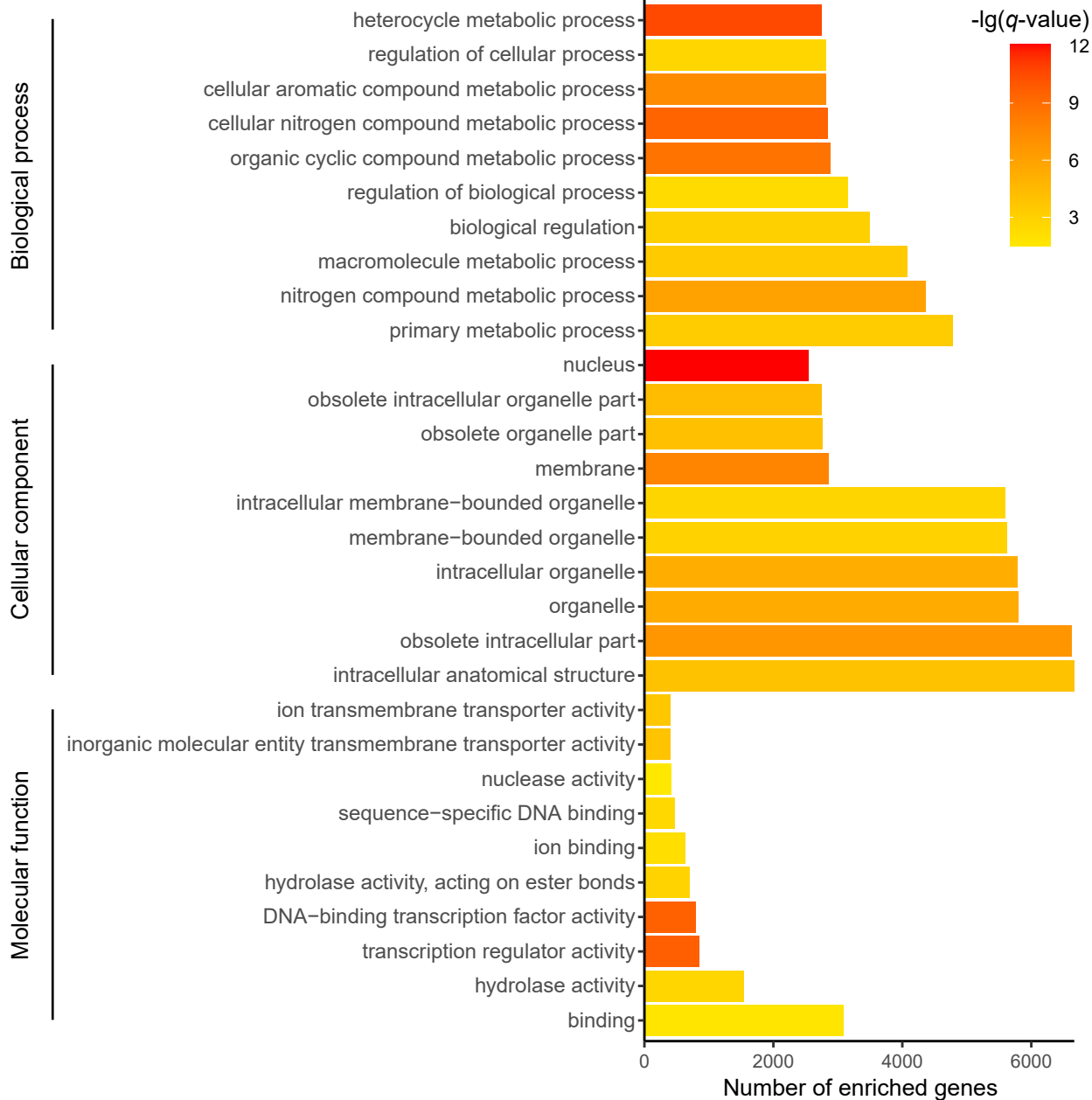

Supplement: Supplementary Figure 1 — Distributions and correlation analysis of Ka, Ks, and Ka/Ks by comparing barley with B. distachyon. (A-C) The frequency distribution of Ka, Ks, and Ka/Ks, respectively. (D) The correlation between Ks (x-axis) and Ka. (E) The correlation between Ks (x-axis) and Ka/Ks. (F) The correlation between Ka (x-axis) and Ka/Ks. (G-I) The box plots of Ka, Ks, and Ka/Ks between expanded and non-expanded genes, respectively. The line in the box is the median value, and the lines at the bottom and top of each box are the first (lower) and third (higher) quartiles. [file DataSheet_1.zip › Supplementary_Figures/Supplementary Figure 11.pdf]

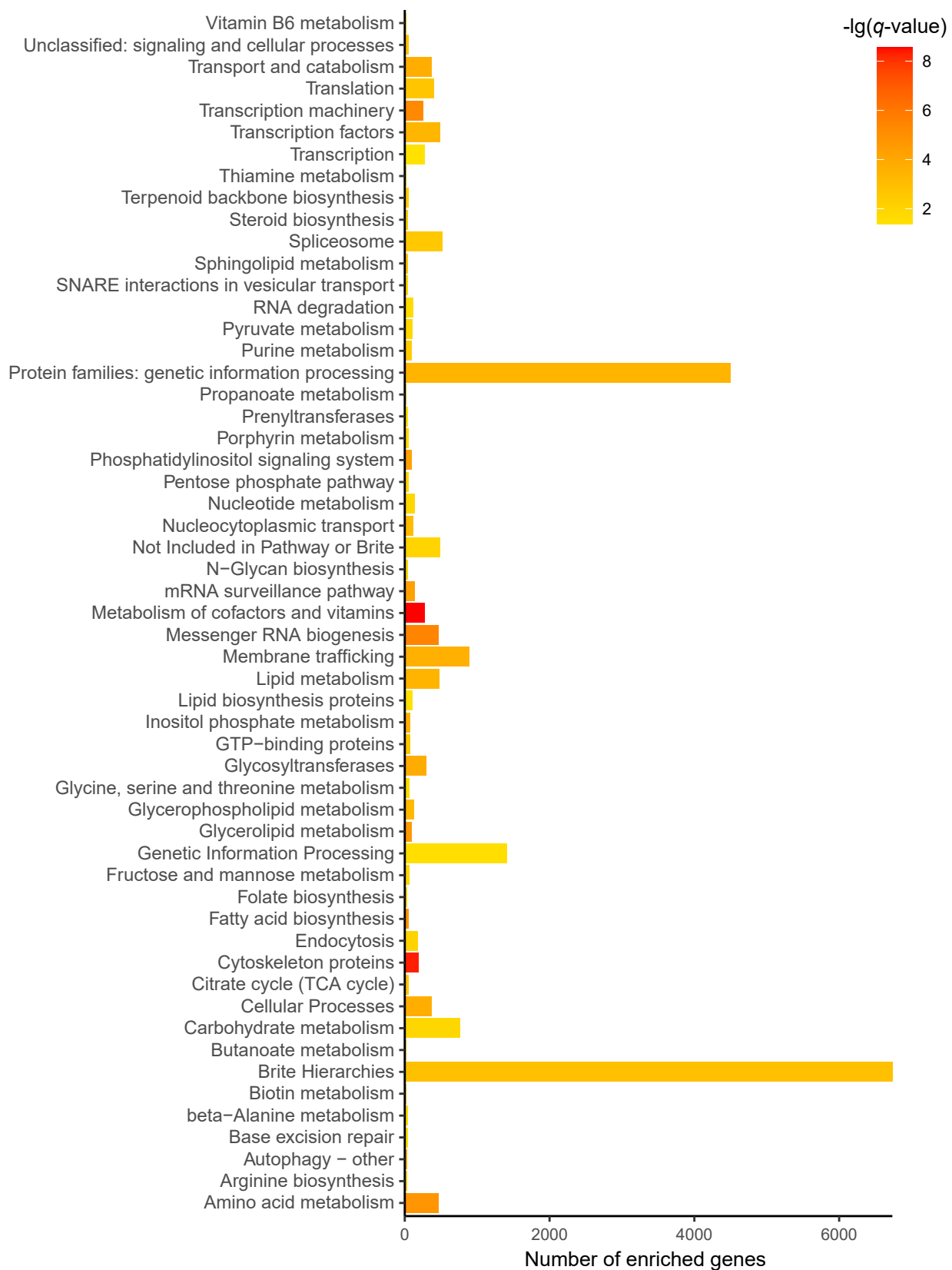

Supplement: Supplementary Figure 1 — Distributions and correlation analysis of Ka, Ks, and Ka/Ks by comparing barley with B. distachyon. (A-C) The frequency distribution of Ka, Ks, and Ka/Ks, respectively. (D) The correlation between Ks (x-axis) and Ka. (E) The correlation between Ks (x-axis) and Ka/Ks. (F) The correlation between Ka (x-axis) and Ka/Ks. (G-I) The box plots of Ka, Ks, and Ka/Ks between expanded and non-expanded genes, respectively. The line in the box is the median value, and the lines at the bottom and top of each box are the first (lower) and third (higher) quartiles. [file DataSheet_1.zip › Supplementary_Figures/Supplementary Figure 12.pdf]

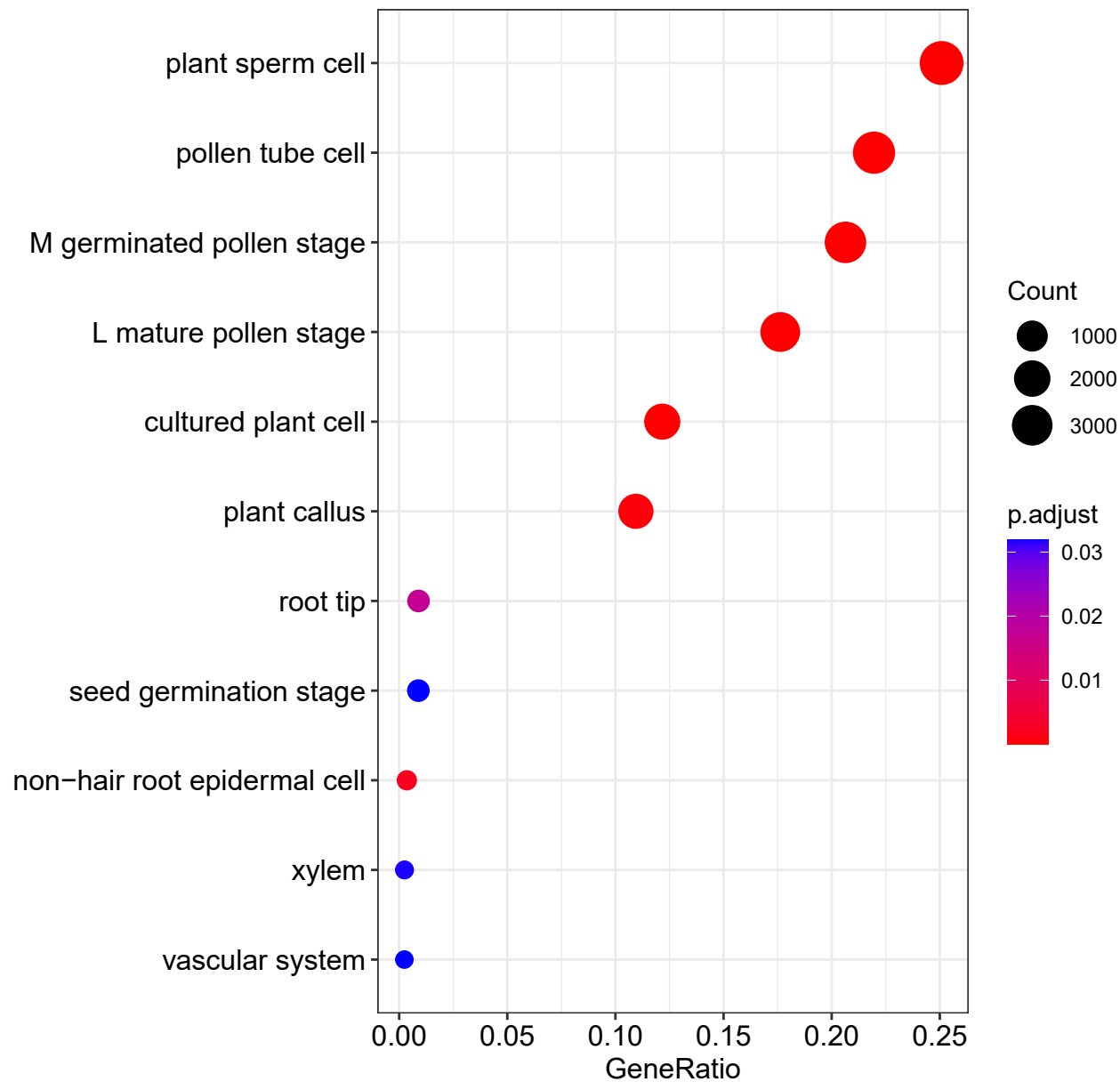

Supplement: Supplementary Figure 1 — Distributions and correlation analysis of Ka, Ks, and Ka/Ks by comparing barley with B. distachyon. (A-C) The frequency distribution of Ka, Ks, and Ka/Ks, respectively. (D) The correlation between Ks (x-axis) and Ka. (E) The correlation between Ks (x-axis) and Ka/Ks. (F) The correlation between Ka (x-axis) and Ka/Ks. (G-I) The box plots of Ka, Ks, and Ka/Ks between expanded and non-expanded genes, respectively. The line in the box is the median value, and the lines at the bottom and top of each box are the first (lower) and third (higher) quartiles. [file DataSheet_1.zip › Supplementary_Figures/Supplementary Figure 13.pdf]

**A**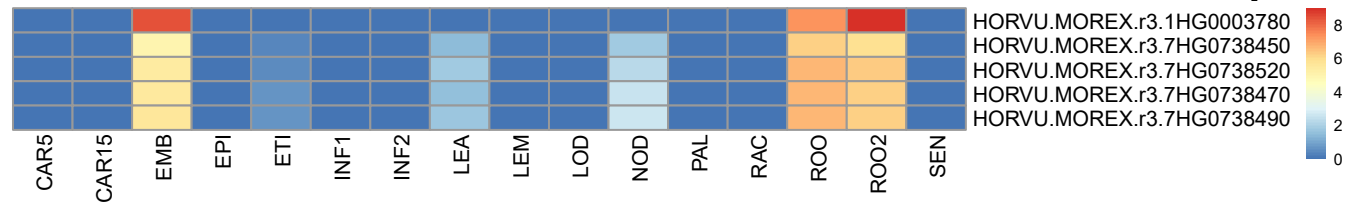**B**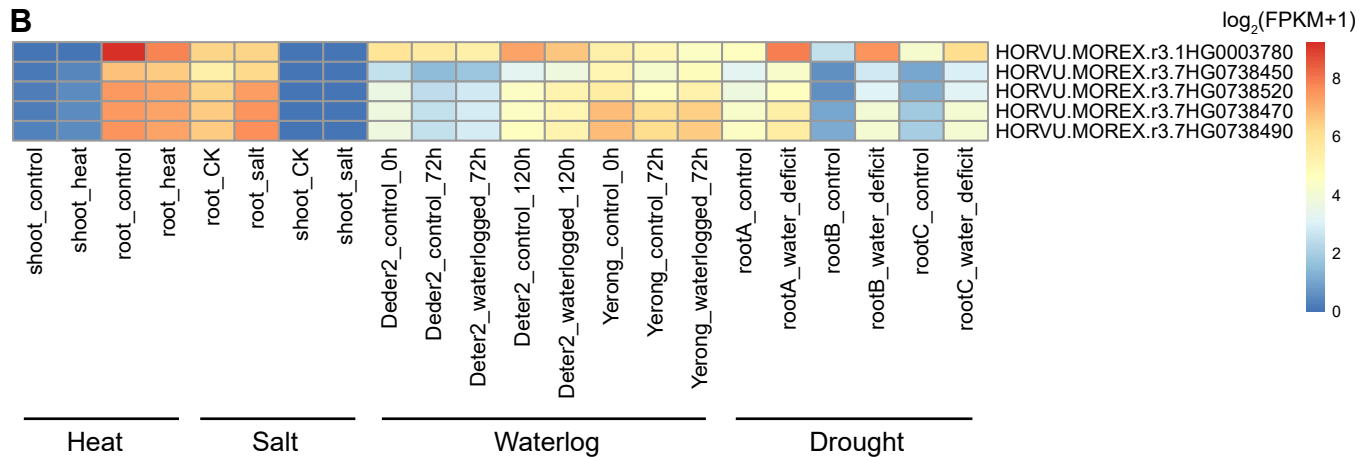

Supplement: Supplementary Figure 1 — Distributions and correlation analysis of Ka, Ks, and Ka/Ks by comparing barley with B. distachyon. (A-C) The frequency distribution of Ka, Ks, and Ka/Ks, respectively. (D) The correlation between Ks (x-axis) and Ka. (E) The correlation between Ks (x-axis) and Ka/Ks. (F) The correlation between Ka (x-axis) and Ka/Ks. (G-I) The box plots of Ka, Ks, and Ka/Ks between expanded and non-expanded genes, respectively. The line in the box is the median value, and the lines at the bottom and top of each box are the first (lower) and third (higher) quartiles. [file DataSheet_1.zip › Supplementary_Figures/Supplementary Figure 14.pdf]

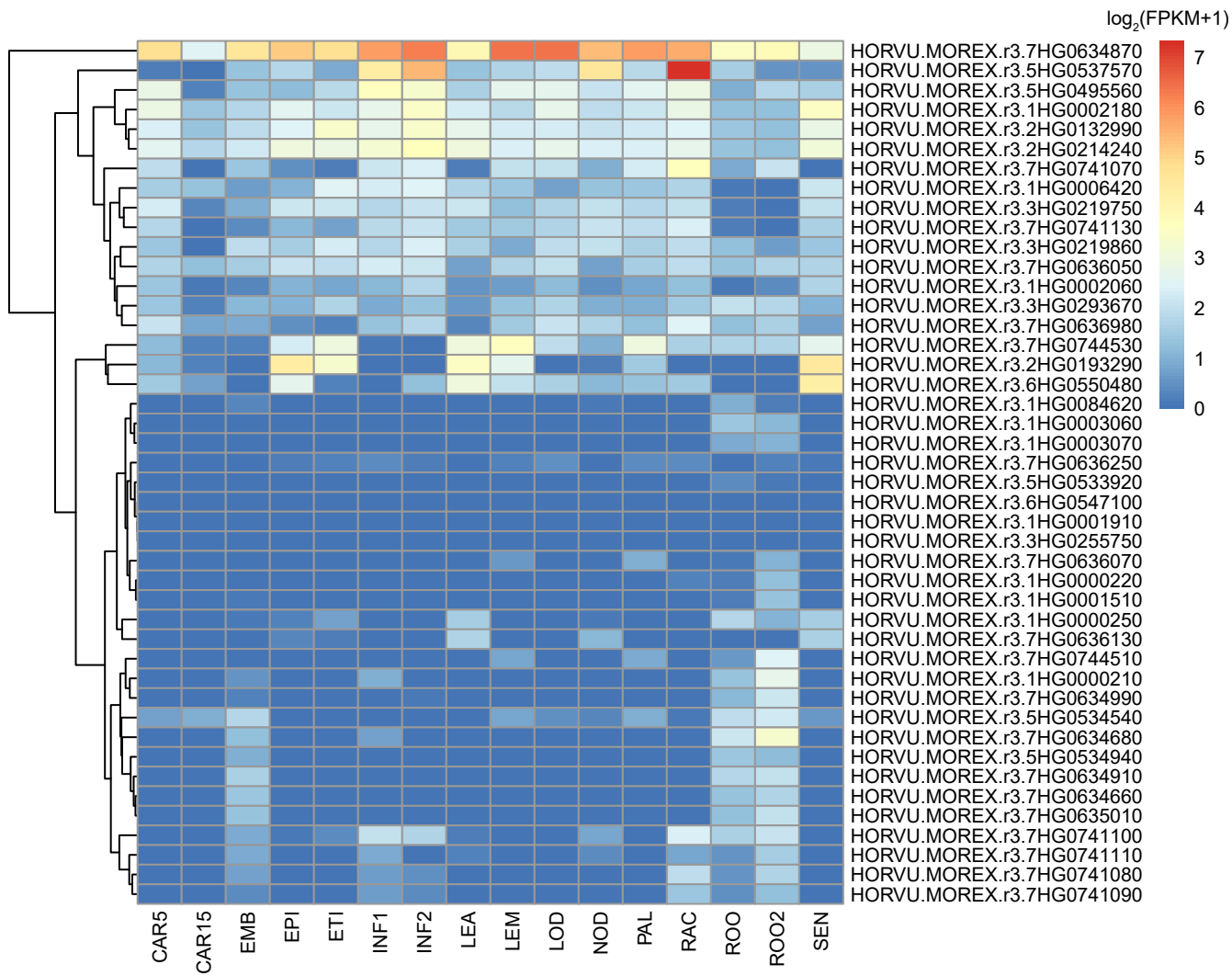

Supplement: Supplementary Figure 1 — Distributions and correlation analysis of Ka, Ks, and Ka/Ks by comparing barley with B. distachyon. (A-C) The frequency distribution of Ka, Ks, and Ka/Ks, respectively. (D) The correlation between Ks (x-axis) and Ka. (E) The correlation between Ks (x-axis) and Ka/Ks. (F) The correlation between Ka (x-axis) and Ka/Ks. (G-I) The box plots of Ka, Ks, and Ka/Ks between expanded and non-expanded genes, respectively. The line in the box is the median value, and the lines at the bottom and top of each box are the first (lower) and third (higher) quartiles. [file DataSheet_1.zip › Supplementary_Figures/Supplementary Figure 15.pdf]

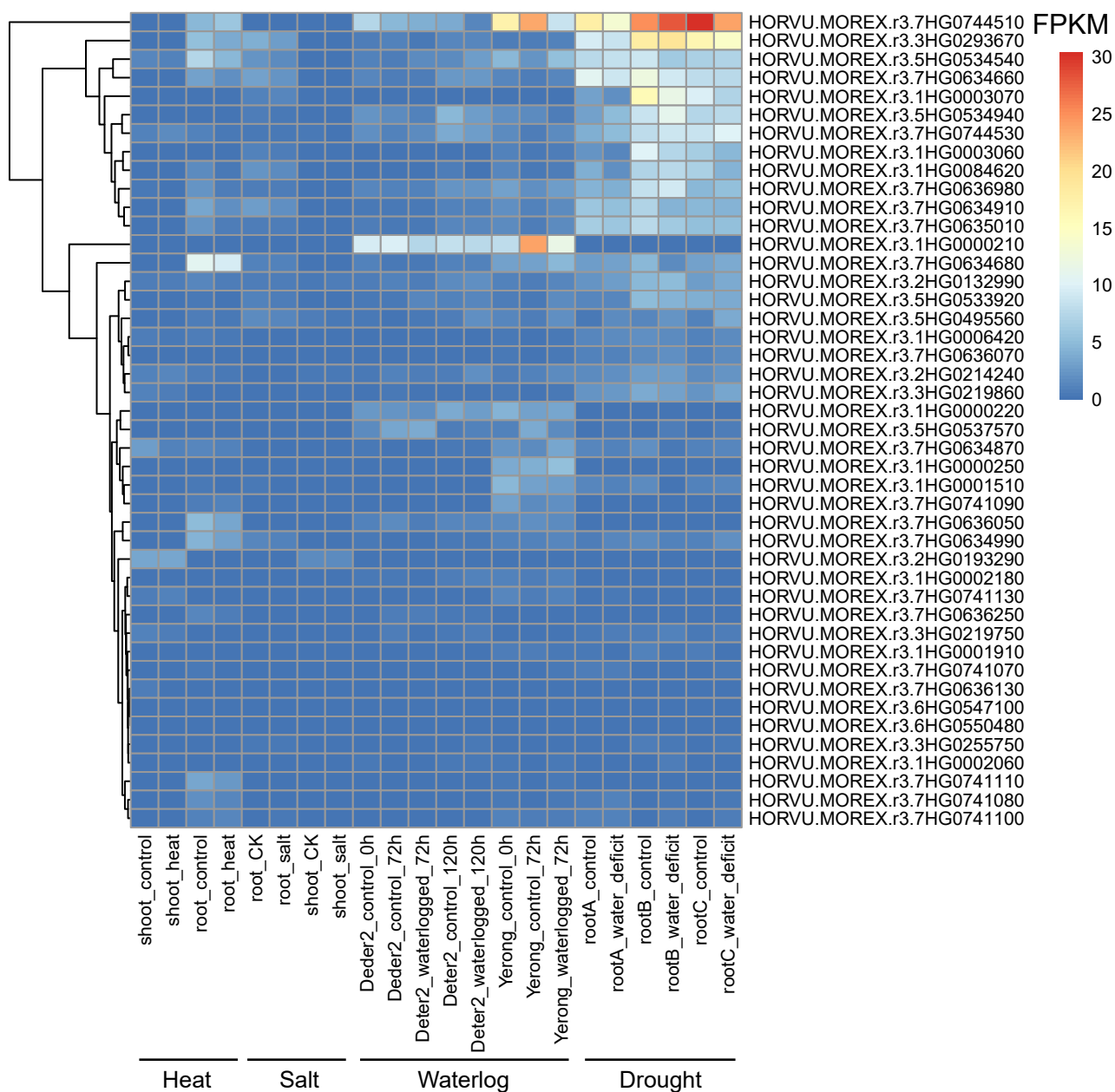

Supplement: Supplementary Figure 1 — Distributions and correlation analysis of Ka, Ks, and Ka/Ks by comparing barley with B. distachyon. (A-C) The frequency distribution of Ka, Ks, and Ka/Ks, respectively. (D) The correlation between Ks (x-axis) and Ka. (E) The correlation between Ks (x-axis) and Ka/Ks. (F) The correlation between Ka (x-axis) and Ka/Ks. (G-I) The box plots of Ka, Ks, and Ka/Ks between expanded and non-expanded genes, respectively. The line in the box is the median value, and the lines at the bottom and top of each box are the first (lower) and third (higher) quartiles. [file DataSheet_1.zip › Supplementary_Figures/Supplementary Figure 16.pdf]

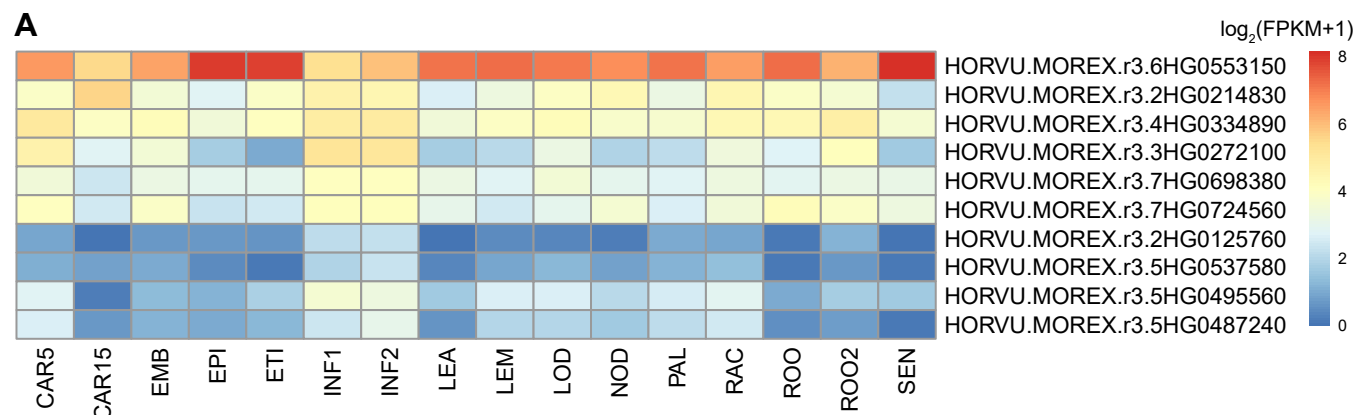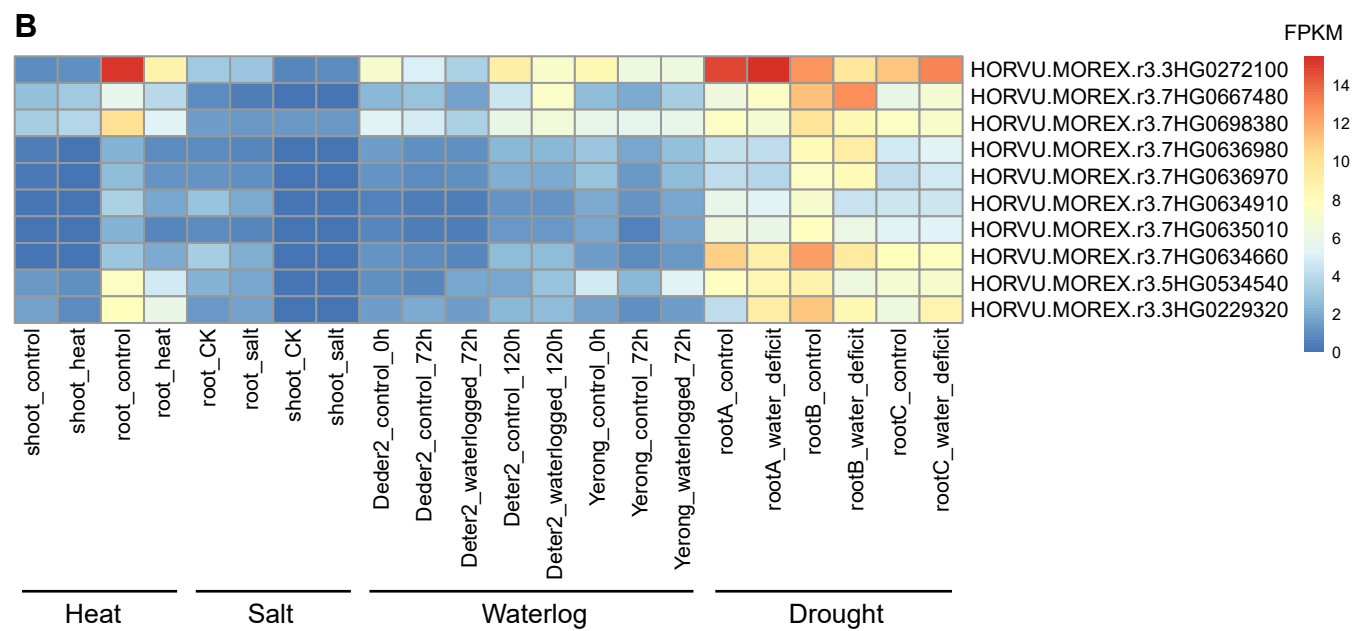

Supplement: Supplementary Figure 1 — Distributions and correlation analysis of Ka, Ks, and Ka/Ks by comparing barley with B. distachyon. (A-C) The frequency distribution of Ka, Ks, and Ka/Ks, respectively. (D) The correlation between Ks (x-axis) and Ka. (E) The correlation between Ks (x-axis) and Ka/Ks. (F) The correlation between Ka (x-axis) and Ka/Ks. (G-I) The box plots of Ka, Ks, and Ka/Ks between expanded and non-expanded genes, respectively. The line in the box is the median value, and the lines at the bottom and top of each box are the first (lower) and third (higher) quartiles. [file DataSheet_1.zip › Supplementary_Figures/Supplementary Figure 17.pdf]

## chr4H

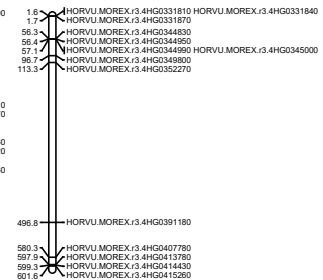

**chr7H**

Supplement: Supplementary Figure 1 — Distributions and correlation analysis of Ka, Ks, and Ka/Ks by comparing barley with B. distachyon. (A-C) The frequency distribution of Ka, Ks, and Ka/Ks, respectively. (D) The correlation between Ks (x-axis) and Ka. (E) The correlation between Ks (x-axis) and Ka/Ks. (F) The correlation between Ka (x-axis) and Ka/Ks. (G-I) The box plots of Ka, Ks, and Ka/Ks between expanded and non-expanded genes, respectively. The line in the box is the median value, and the lines at the bottom and top of each box are the first (lower) and third (higher) quartiles. [file DataSheet_1.zip › Supplementary_Figures/Supplementary Figure 19.pdf]

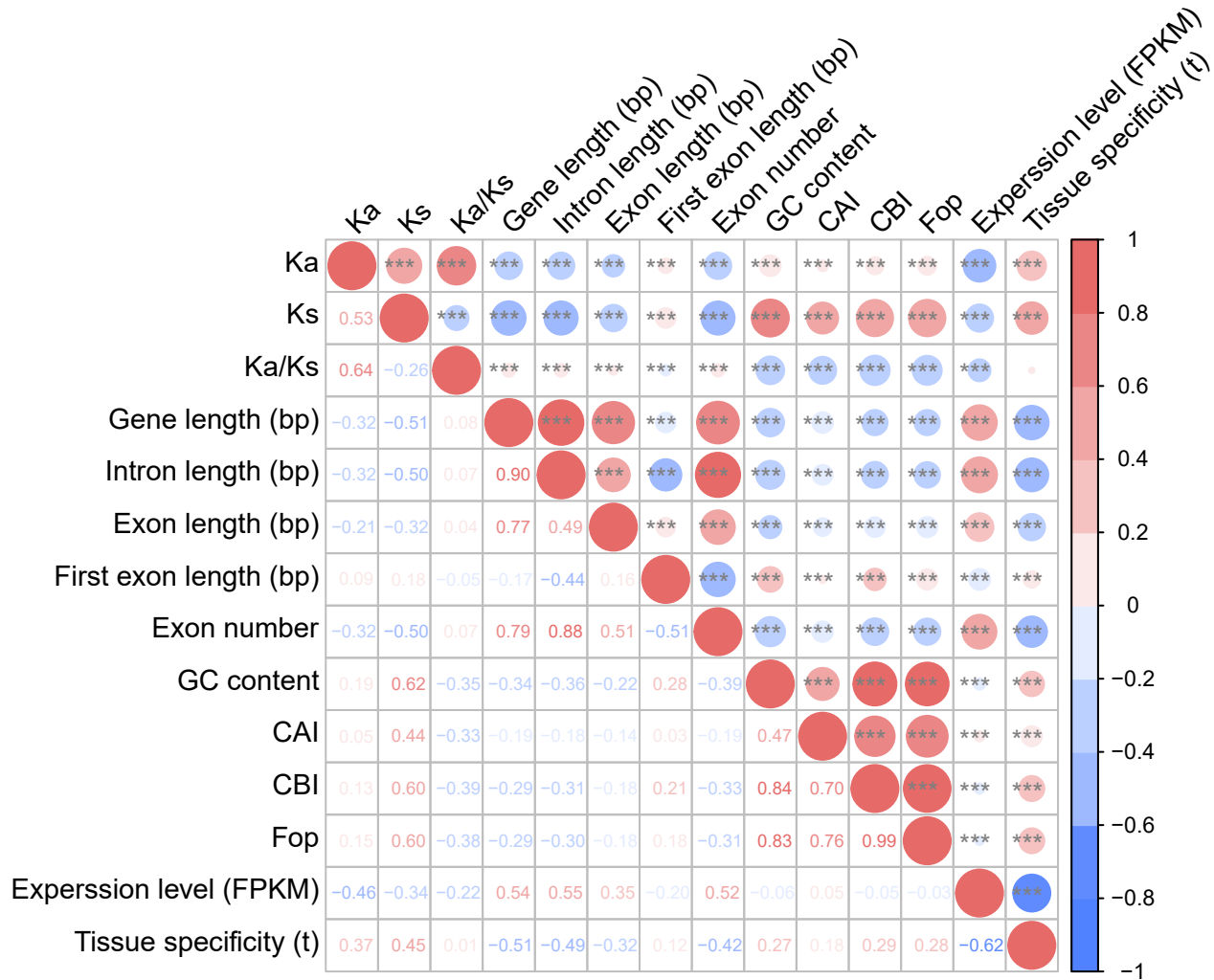

Supplement: Supplementary Figure 1 — Distributions and correlation analysis of Ka, Ks, and Ka/Ks by comparing barley with B. distachyon. (A-C) The frequency distribution of Ka, Ks, and Ka/Ks, respectively. (D) The correlation between Ks (x-axis) and Ka. (E) The correlation between Ks (x-axis) and Ka/Ks. (F) The correlation between Ka (x-axis) and Ka/Ks. (G-I) The box plots of Ka, Ks, and Ka/Ks between expanded and non-expanded genes, respectively. The line in the box is the median value, and the lines at the bottom and top of each box are the first (lower) and third (higher) quartiles. [file DataSheet_1.zip › Supplementary_Figures/Supplementary Figure 2.pdf]

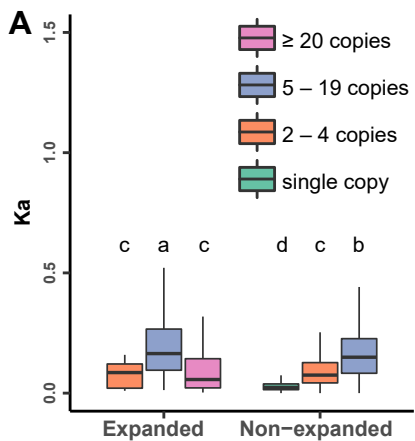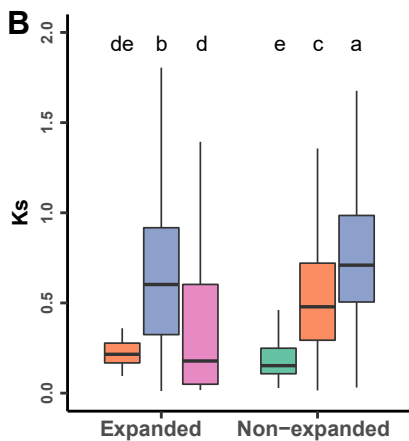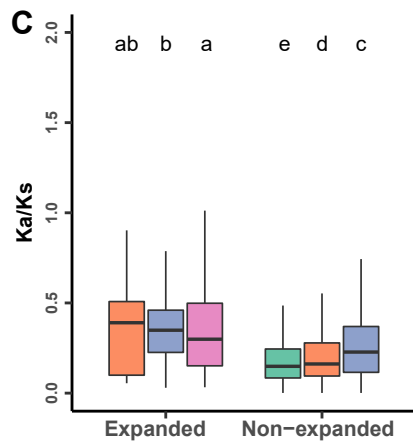

Supplement: Supplementary Figure 1 — Distributions and correlation analysis of Ka, Ks, and Ka/Ks by comparing barley with B. distachyon. (A-C) The frequency distribution of Ka, Ks, and Ka/Ks, respectively. (D) The correlation between Ks (x-axis) and Ka. (E) The correlation between Ks (x-axis) and Ka/Ks. (F) The correlation between Ka (x-axis) and Ka/Ks. (G-I) The box plots of Ka, Ks, and Ka/Ks between expanded and non-expanded genes, respectively. The line in the box is the median value, and the lines at the bottom and top of each box are the first (lower) and third (higher) quartiles. [file DataSheet_1.zip › Supplementary_Figures/Supplementary Figure 3.pdf]

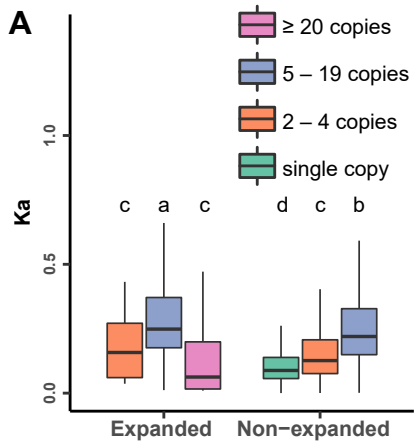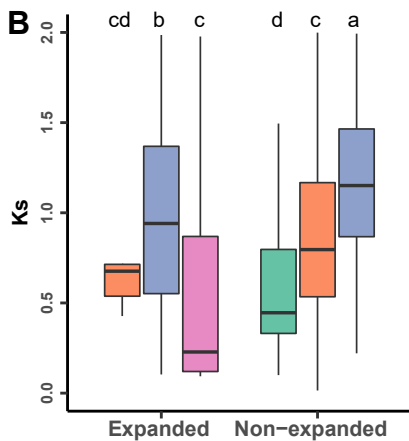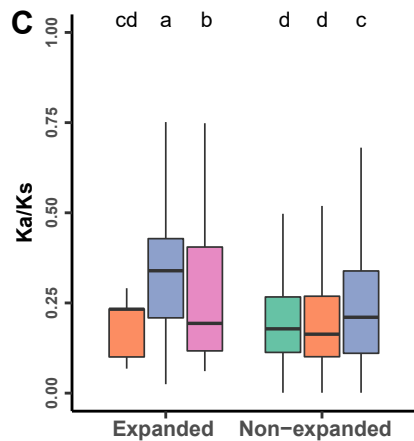

Supplement: Supplementary Figure 1 — Distributions and correlation analysis of Ka, Ks, and Ka/Ks by comparing barley with B. distachyon. (A-C) The frequency distribution of Ka, Ks, and Ka/Ks, respectively. (D) The correlation between Ks (x-axis) and Ka. (E) The correlation between Ks (x-axis) and Ka/Ks. (F) The correlation between Ka (x-axis) and Ka/Ks. (G-I) The box plots of Ka, Ks, and Ka/Ks between expanded and non-expanded genes, respectively. The line in the box is the median value, and the lines at the bottom and top of each box are the first (lower) and third (higher) quartiles. [file DataSheet_1.zip › Supplementary_Figures/Supplementary Figure 4.pdf]

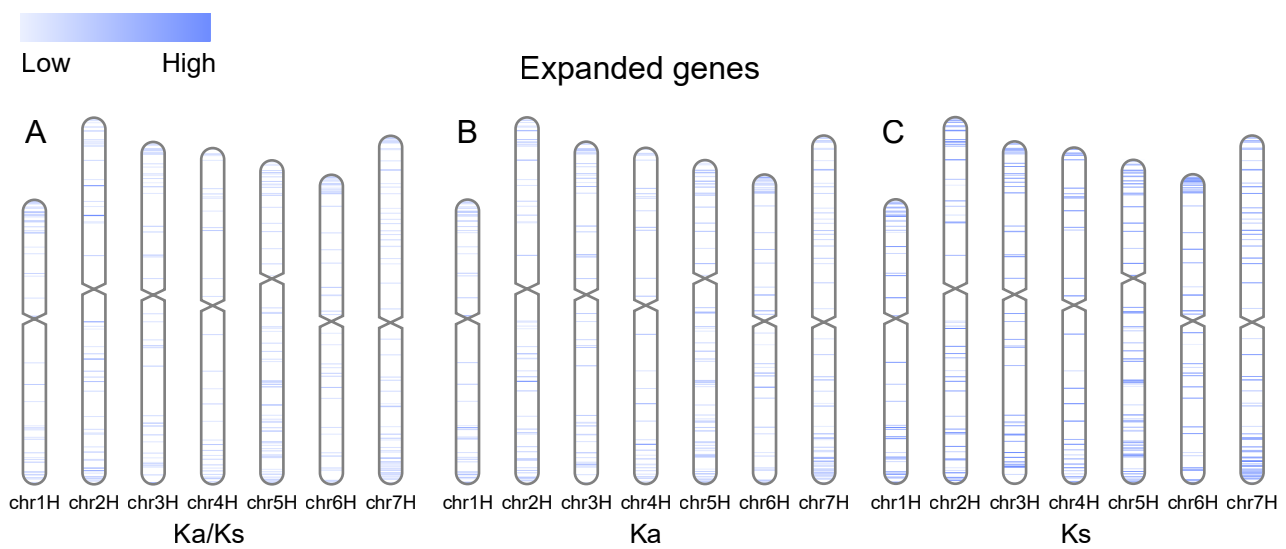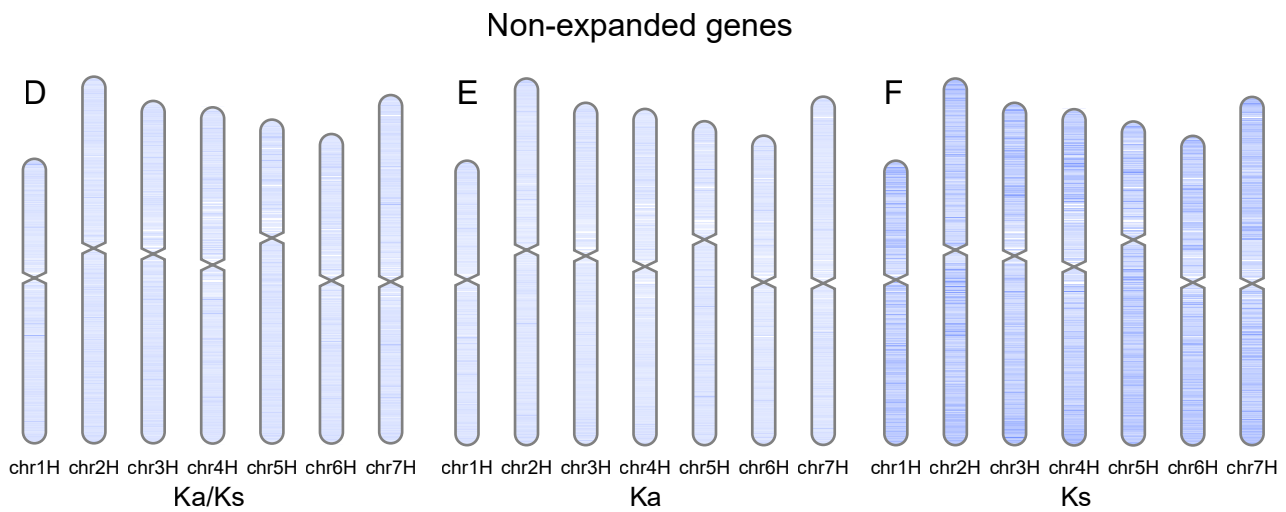

Supplement: Supplementary Figure 1 — Distributions and correlation analysis of Ka, Ks, and Ka/Ks by comparing barley with B. distachyon. (A-C) The frequency distribution of Ka, Ks, and Ka/Ks, respectively. (D) The correlation between Ks (x-axis) and Ka. (E) The correlation between Ks (x-axis) and Ka/Ks. (F) The correlation between Ka (x-axis) and Ka/Ks. (G-I) The box plots of Ka, Ks, and Ka/Ks between expanded and non-expanded genes, respectively. The line in the box is the median value, and the lines at the bottom and top of each box are the first (lower) and third (higher) quartiles. [file DataSheet_1.zip › Supplementary_Figures/Supplementary Figure 5.pdf]

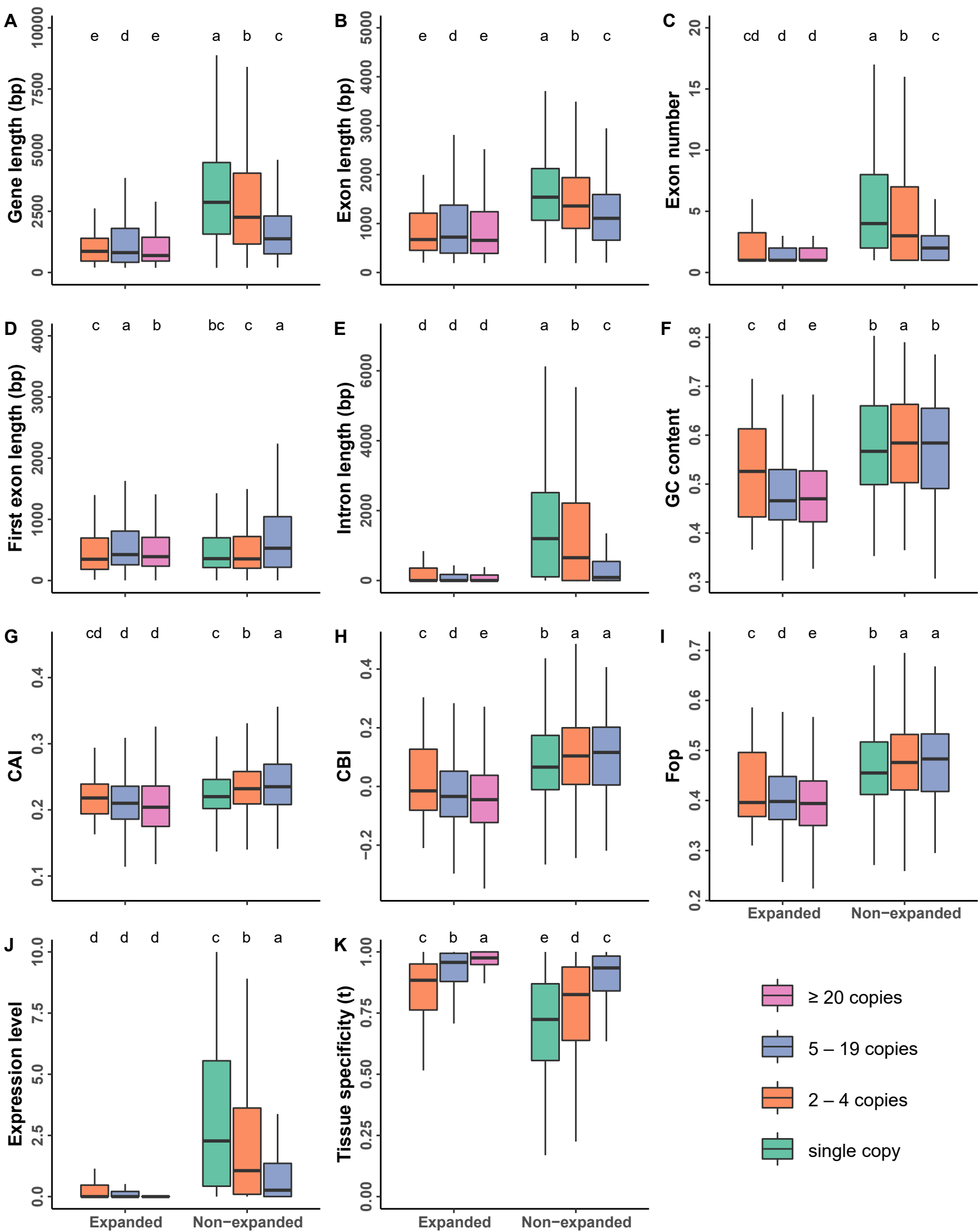

Supplement: Supplementary Figure 1 — Distributions and correlation analysis of Ka, Ks, and Ka/Ks by comparing barley with B. distachyon. (A-C) The frequency distribution of Ka, Ks, and Ka/Ks, respectively. (D) The correlation between Ks (x-axis) and Ka. (E) The correlation between Ks (x-axis) and Ka/Ks. (F) The correlation between Ka (x-axis) and Ka/Ks. (G-I) The box plots of Ka, Ks, and Ka/Ks between expanded and non-expanded genes, respectively. The line in the box is the median value, and the lines at the bottom and top of each box are the first (lower) and third (higher) quartiles. [file DataSheet_1.zip › Supplementary_Figures/Supplementary Figure 6.pdf]

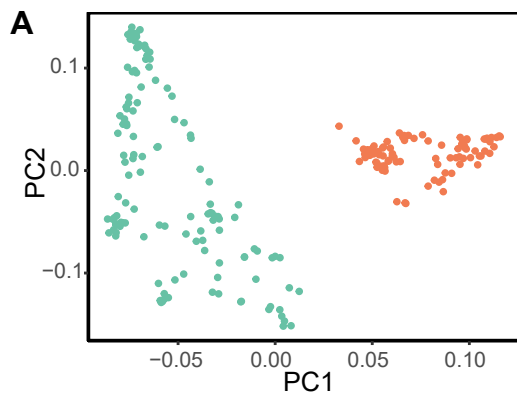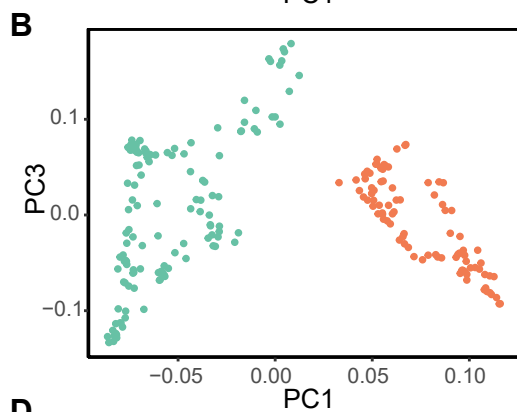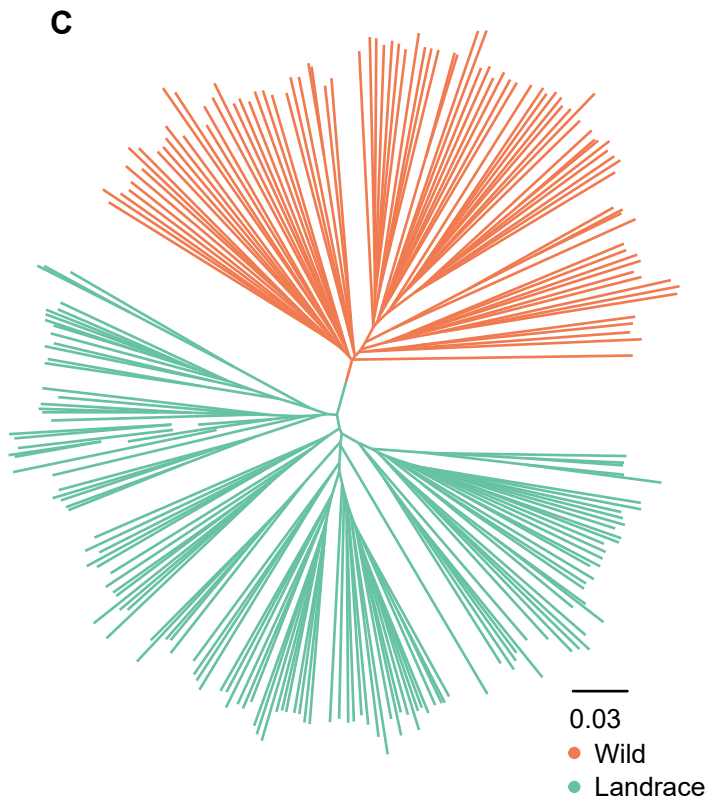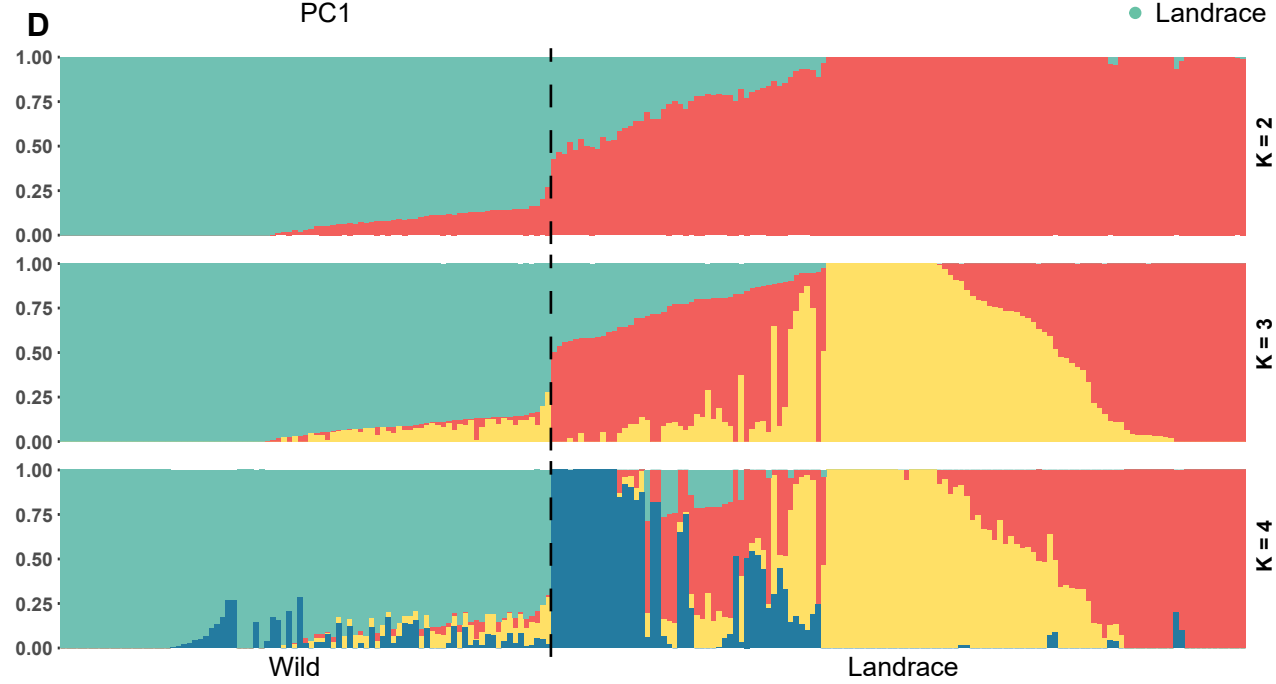

Supplement: Supplementary Figure 1 — Distributions and correlation analysis of Ka, Ks, and Ka/Ks by comparing barley with B. distachyon. (A-C) The frequency distribution of Ka, Ks, and Ka/Ks, respectively. (D) The correlation between Ks (x-axis) and Ka. (E) The correlation between Ks (x-axis) and Ka/Ks. (F) The correlation between Ka (x-axis) and Ka/Ks. (G-I) The box plots of Ka, Ks, and Ka/Ks between expanded and non-expanded genes, respectively. The line in the box is the median value, and the lines at the bottom and top of each box are the first (lower) and third (higher) quartiles. [file DataSheet_1.zip › Supplementary_Figures/Supplementary Figure 7.pdf]

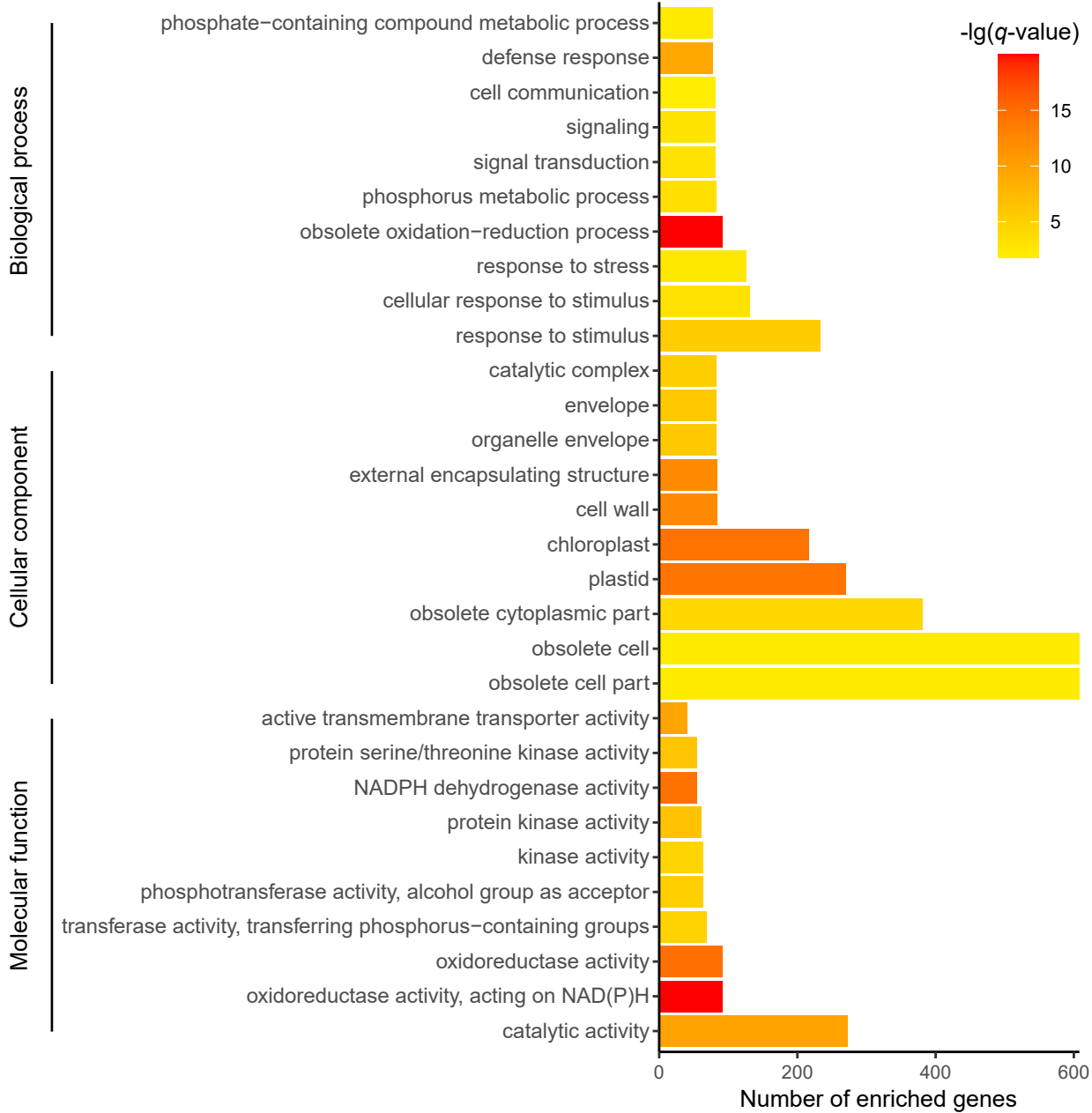

Supplement: Supplementary Figure 1 — Distributions and correlation analysis of Ka, Ks, and Ka/Ks by comparing barley with B. distachyon. (A-C) The frequency distribution of Ka, Ks, and Ka/Ks, respectively. (D) The correlation between Ks (x-axis) and Ka. (E) The correlation between Ks (x-axis) and Ka/Ks. (F) The correlation between Ka (x-axis) and Ka/Ks. (G-I) The box plots of Ka, Ks, and Ka/Ks between expanded and non-expanded genes, respectively. The line in the box is the median value, and the lines at the bottom and top of each box are the first (lower) and third (higher) quartiles. [file DataSheet_1.zip › Supplementary_Figures/Supplementary Figure 8.pdf]

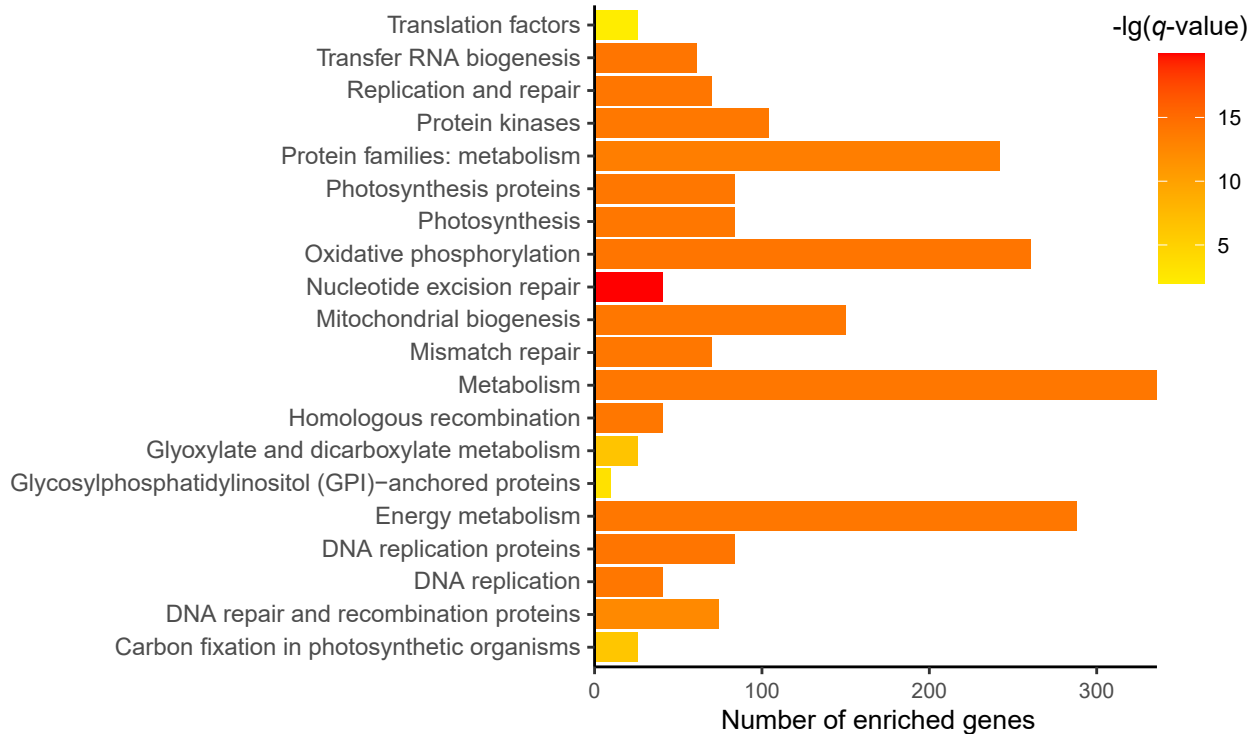

Supplement: Supplementary Figure 1 — Distributions and correlation analysis of Ka, Ks, and Ka/Ks by comparing barley with B. distachyon. (A-C) The frequency distribution of Ka, Ks, and Ka/Ks, respectively. (D) The correlation between Ks (x-axis) and Ka. (E) The correlation between Ks (x-axis) and Ka/Ks. (F) The correlation between Ka (x-axis) and Ka/Ks. (G-I) The box plots of Ka, Ks, and Ka/Ks between expanded and non-expanded genes, respectively. The line in the box is the median value, and the lines at the bottom and top of each box are the first (lower) and third (higher) quartiles. [file DataSheet_1.zip › Supplementary_Figures/Supplementary Figure 9.pdf]
