# Supplementary material for: The evolutionary patterns, expression profiles, and genetic diversity of expanded genes in barley
Source: Front Plant Sci. 2023 Apr 26;14:1168124. doi: 10.3389/fpls.2023.1168124 (PMC10171312; doi:10.3389/fpls.2023.1168124)
Supplement: Supplementary Figure 1 — Distributions and correlation analysis of Ka, Ks, and Ka/Ks by comparing barley with B. distachyon. (A-C) The frequency distribution of Ka, Ks, and Ka/Ks, respectively. (D) The correlation between Ks (x-axis) and Ka. (E) The correlation between Ks (x-axis) and Ka/Ks. (F) The correlation between Ka (x-axis) and Ka/Ks. (G-I) The box plots of Ka, Ks, and Ka/Ks between expanded and non-expanded genes, respectively. The line in the box is the median value, and the lines at the bottom and top of each box are the first (lower) and third (higher) quartiles. [file Supplementary Figure 18.pdf › Supplementary_Figures/Supplementary Figure 18.pdf]

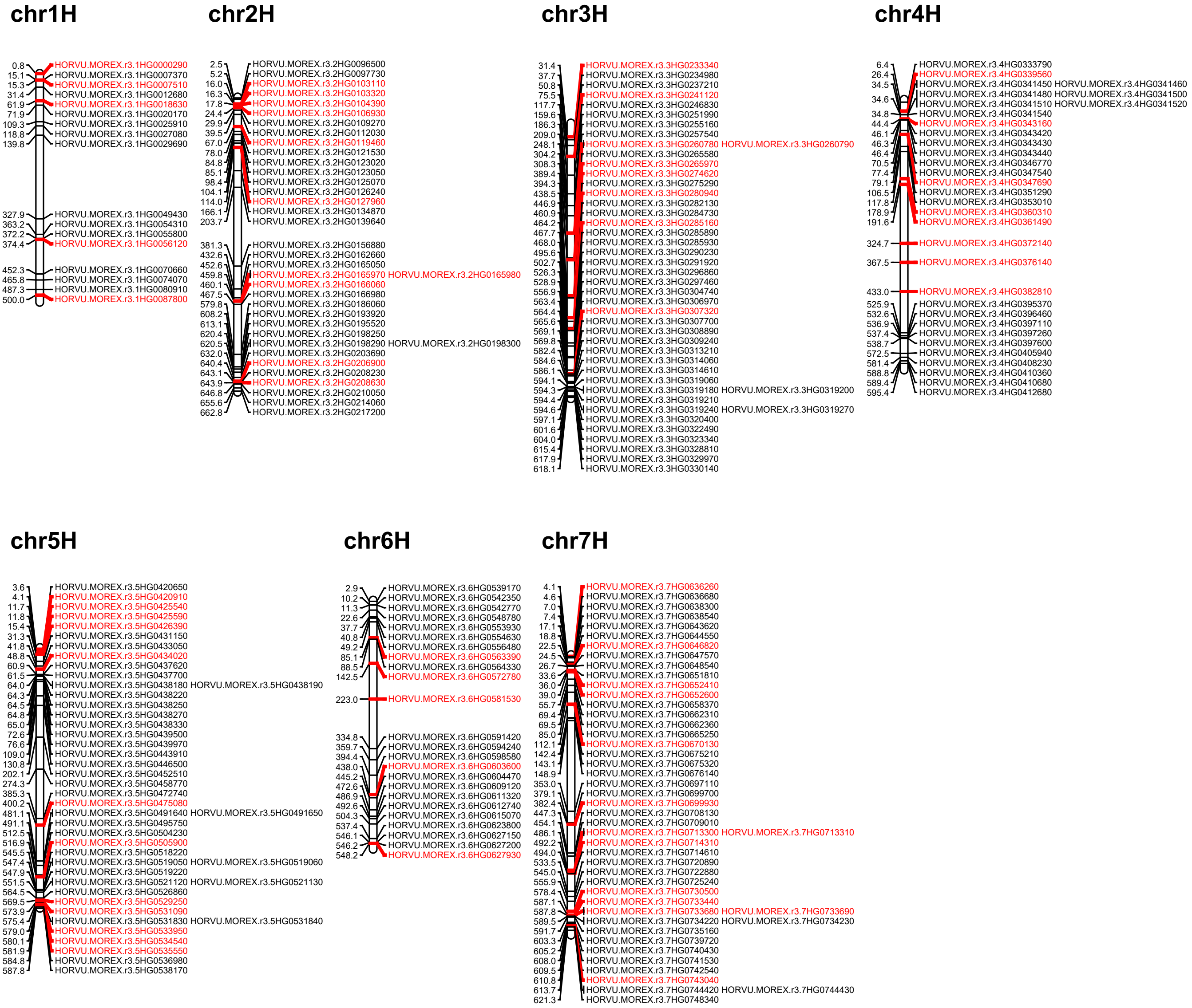
chr1H
0.8 HORVU.MOREX.r3.1HG0000290
15.1 HORVU.MOREX.r3.1HG0007370
15.3 HORVU.MOREX.r3.1HG0007510
31.4 HORVU.MOREX.r3.1HG0012680
61.9 HORVU.MOREX.r3.1HG0018630
71.9 HORVU.MOREX.r3.1HG0020170
109.3 HORVU.MOREX.r3.1HG0025910
118.8 HORVU.MOREX.r3.1HG0027080
139.8 HORVU.MOREX.r3.1HG0029690
327.9 HORVU.MOREX.r3.1HG0049430
363.2 HORVU.MOREX.r3.1HG0054310
372.2 HORVU.MOREX.r3.1HG0055800
374.4 HORVU.MOREX.r3.1HG0056120
452.3 HORVU.MOREX.r3.1HG0070660
465.8 HORVU.MOREX.r3.1HG0074070
487.3 HORVU.MOREX.r3.1HG0080910
500.0 HORVU.MOREX.r3.1HG0087800
chr2H
2.5 HORVU.MOREX.r3.2HG0096500
5.2 HORVU.MOREX.r3.2HG0097730
16.0 HORVU.MOREX.r3.2HG0103110
16.3 HORVU.MOREX.r3.2HG0103320
17.8 HORVU.MOREX.r3.2HG0104390
24.4 HORVU.MOREX.r3.2HG0106930
29.9 HORVU.MOREX.r3.2HG0109270
39.5 HORVU.MOREX.r3.2HG0112030
67.0 HORVU.MOREX.r3.2HG0119460
78.0 HORVU.MOREX.r3.2HG0121530
84.8 HORVU.MOREX.r3.2HG0123020
85.1 HORVU.MOREX.r3.2HG0123050
98.4 HORVU.MOREX.r3.2HG0125070
104.1 HORVU.MOREX.r3.2HG0126240
114.0 HORVU.MOREX.r3.2HG0127960
166.1 HORVU.MOREX.r3.2HG0134870
203.7 HORVU.MOREX.r3.2HG0139640
381.3 HORVU.MOREX.r3.2HG0156880
432.6 HORVU.MOREX.r3.2HG0162660
452.6 HORVU.MOREX.r3.2HG0165050
459.8 HORVU.MOREX.r3.2HG0165970 HORVU.MOREX.r3.2HG0165980
460.1 HORVU.MOREX.r3.2HG0166060
467.5 HORVU.MOREX.r3.2HG0166980
579.8 HORVU.MOREX.r3.2HG0186060
608.2 HORVU.MOREX.r3.2HG0193920
613.1 HORVU.MOREX.r3.2HG0195520
620.4 HORVU.MOREX.r3.2HG0198250
620.5 HORVU.MOREX.r3.2HG0198290 HORVU.MOREX.r3.2HG0198300
632.0 HORVU.MOREX.r3.2HG0203690
640.4 HORVU.MOREX.r3.2HG0206900
643.1 HORVU.MOREX.r3.2HG0208230
643.9 HORVU.MOREX.r3.2HG0208630
646.8 HORVU.MOREX.r3.2HG0210050
655.6 HORVU.MOREX.r3.2HG0214060
662.8 HORVU.MOREX.r3.2HG0217200
chr3H
31.4 HORVU.MOREX.r3.3HG0233340
37.7 HORVU.MOREX.r3.3HG0234980
50.8 HORVU.MOREX.r3.3HG0237210
75.5 HORVU.MOREX.r3.3HG0241120
117.7 HORVU.MOREX.r3.3HG0246830
159.6 HORVU.MOREX.r3.3HG0251990
186.3 HORVU.MOREX.r3.3HG0255160
209.0 HORVU.MOREX.r3.3HG0257540
248.1 HORVU.MOREX.r3.3HG0260780 HORVU.MOREX.r3.3HG0260790
304.2 HORVU.MOREX.r3.3HG0265580
308.3 HORVU.MOREX.r3.3HG0265970
389.4 HORVU.MOREX.r3.3HG0274620
394.3 HORVU.MOREX.r3.3HG0275290
438.5 HORVU.MOREX.r3.3HG0280940
446.9 HORVU.MOREX.r3.3HG0282130
460.9 HORVU.MOREX.r3.3HG0284730
464.2 HORVU.MOREX.r3.3HG0285160
467.7 HORVU.MOREX.r3.3HG0285890
468.0 HORVU.MOREX.r3.3HG0285930
495.6 HORVU.MOREX.r3.3HG0290230
502.7 HORVU.MOREX.r3.3HG0291920
526.3 HORVU.MOREX.r3.3HG0296860
528.9 HORVU.MOREX.r3.3HG0297460
556.9 HORVU.MOREX.r3.3HG0304740
563.4 HORVU.MOREX.r3.3HG0306970
564.4 HORVU.MOREX.r3.3HG0307320
565.6 HORVU.MOREX.r3.3HG0307700
569.1 HORVU.MOREX.r3.3HG0308890
569.8 HORVU.MOREX.r3.3HG0309240
582.4 HORVU.MOREX.r3.3HG0313210
584.6 HORVU.MOREX.r3.3HG0314060
586.1 HORVU.MOREX.r3.3HG0314610
594.1 HORVU.MOREX.r3.3HG0319060
594.3 HORVU.MOREX.r3.3HG0319180 HORVU.MOREX.r3.3HG0319200
594.4 HORVU.MOREX.r3.3HG0319210
594.6 HORVU.MOREX.r3.3HG0319240 HORVU.MOREX.r3.3HG0319270
597.1 HORVU.MOREX.r3.3HG0320400
601.6 HORVU.MOREX.r3.3HG0322490
604.0 HORVU.MOREX.r3.3HG0323340
615.4 HORVU.MOREX.r3.3HG0328810
617.9 HORVU.MOREX.r3.3HG0329970
618.1 HORVU.MOREX.r3.3HG0330140
chr4H
6.4 HORVU.MOREX.r3.4HG0333790
26.4 HORVU.MOREX.r3.4HG0339560
34.5 HORVU.MOREX.r3.4HG0341450 HORVU.MOREX.r3.4HG0341460
34.6 HORVU.MOREX.r3.4HG0341480 HORVU.MOREX.r3.4HG0341500
HORVU.MOREX.r3.4HG0341510 HORVU.MOREX.r3.4HG0341520
34.8 HORVU.MOREX.r3.4HG0341540
44.4 HORVU.MOREX.r3.4HG0343160
46.1 HORVU.MOREX.r3.4HG0343420
46.3 HORVU.MOREX.r3.4HG0343430
46.4 HORVU.MOREX.r3.4HG0343440
70.5 HORVU.MOREX.r3.4HG0346770
77.4 HORVU.MOREX.r3.4HG0347540
79.1 HORVU.MOREX.r3.4HG0347690
106.5 HORVU.MOREX.r3.4HG0351290
117.8 HORVU.MOREX.r3.4HG0353010
178.9 HORVU.MOREX.r3.4HG0360310
191.6 HORVU.MOREX.r3.4HG0361490
324.7 HORVU.MOREX.r3.4HG0372140
367.5 HORVU.MOREX.r3.4HG0376140
433.0 HORVU.MOREX.r3.4HG0382810
525.9 HORVU.MOREX.r3.4HG0395370
532.6 HORVU.MOREX.r3.4HG0396460
536.9 HORVU.MOREX.r3.4HG0397110
537.4 HORVU.MOREX.r3.4HG0397260
538.7 HORVU.MOREX.r3.4HG0397600
572.5 HORVU.MOREX.r3.4HG0405940
581.4 HORVU.MOREX.r3.4HG0408230
588.8 HORVU.MOREX.r3.4HG0410360
589.4 HORVU.MOREX.r3.4HG0410680
595.4 HORVU.MOREX.r3.4HG0412680
chr5H
3.6 HORVU.MOREX.r3.5HG0420650
4.1 HORVU.MOREX.r3.5HG0420910
11.7 HORVU.MOREX.r3.5HG0425540
11.8 HORVU.MOREX.r3.5HG0425590
15.4 HORVU.MOREX.r3.5HG0426390
31.3 HORVU.MOREX.r3.5HG0431150
41.8 HORVU.MOREX.r3.5HG0433050
48.8 HORVU.MOREX.r3.5HG0434020
60.9 HORVU.MOREX.r3.5HG0437620
61.5 HORVU.MOREX.r3.5HG0437700
64.0 HORVU.MOREX.r3.5HG0438180 HORVU.MOREX.r3.5HG0438190
64.3 HORVU.MOREX.r3.5HG0438220
64.5 HORVU.MOREX.r3.5HG0438250
64.8 HORVU.MOREX.r3.5HG0438270
65.0 HORVU.MOREX.r3.5HG0438330
72.6 HORVU.MOREX.r3.5HG0439500
76.6 HORVU.MOREX.r3.5HG0439970
109.0 HORVU.MOREX.r3.5HG0443910
130.8 HORVU.MOREX.r3.5HG0446500
202.1 HORVU.MOREX.r3.5HG0452510
274.3 HORVU.MOREX.r3.5HG0458770
385.3 HORVU.MOREX.r3.5HG0472740
400.2 HORVU.MOREX.r3.5HG0475080
481.1 HORVU.MOREX.r3.5HG0491640 HORVU.MOREX.r3.5HG0491650
491.1 HORVU.MOREX.r3.5HG0495750
512.5 HORVU.MOREX.r3.5HG0504230
516.9 HORVU.MOREX.r3.5HG0505900
545.5 HORVU.MOREX.r3.5HG0518220
547.4 HORVU.MOREX.r3.5HG0519050 HORVU.MOREX.r3.5HG0519060
547.9 HORVU.MOREX.r3.5HG0519220
551.5 HORVU.MOREX.r3.5HG0521120 HORVU.MOREX.r3.5HG0521130
564.5 HORVU.MOREX.r3.5HG0526860
569.5 HORVU.MOREX.r3.5HG0529250
573.9 HORVU.MOREX.r3.5HG0531090
575.4 HORVU.MOREX.r3.5HG0531830 HORVU.MOREX.r3.5HG0531840
579.0 HORVU.MOREX.r3.5HG0533950
580.1 HORVU.MOREX.r3.5HG0534540
581.9 HORVU.MOREX.r3.5HG0535550
584.8 HORVU.MOREX.r3.5HG0536980
587.8 HORVU.MOREX.r3.5HG0538170
chr6H
2.9 HORVU.MOREX.r3.6HG0539170
10.2 HORVU.MOREX.r3.6HG0542350
11.3 HORVU.MOREX.r3.6HG0542770
22.6 HORVU.MOREX.r3.6HG0548780
37.7 HORVU.MOREX.r3.6HG0553930
40.8 HORVU.MOREX.r3.6HG0554630
49.2 HORVU.MOREX.r3.6HG0556480
85.1 HORVU.MOREX.r3.6HG0563390
88.5 HORVU.MOREX.r3.6HG0564330
142.5 HORVU.MOREX.r3.6HG0572780
223.0 HORVU.MOREX.r3.6HG0581530
334.8 HORVU.MOREX.r3.6HG0591420
359.7 HORVU.MOREX.r3.6HG0594240
394.4 HORVU.MOREX.r3.6HG0598580
438.0 HORVU.MOREX.r3.6HG0603600
445.2 HORVU.MOREX.r3.6HG0604470
472.6 HORVU.MOREX.r3.6HG0609120
486.9 HORVU.MOREX.r3.6HG0611320
492.6 HORVU.MOREX.r3.6HG0612740
504.3 HORVU.MOREX.r3.6HG0615070
537.4 HORVU.MOREX.r3.6HG0623800
546.1 HORVU.MOREX.r3.6HG0627150
546.2 HORVU.MOREX.r3.6HG0627200
548.2 HORVU.MOREX.r3.6HG0627930
chr7H
4.1 HORVU.MOREX.r3.7HG0636260
4.6 HORVU.MOREX.r3.7HG0636680
7.0 HORVU.MOREX.r3.7HG0638300
7.4 HORVU.MOREX.r3.7HG0638540
17.1 HORVU.MOREX.r3.7HG0643620
18.8 HORVU.MOREX.r3.7HG0644550
22.5 HORVU.MOREX.r3.7HG0646820
24.5 HORVU.MOREX.r3.7HG0647570
26.7 HORVU.MOREX.r3.7HG0648540
33.6 HORVU.MOREX.r3.7HG0651810
36.0 HORVU.MOREX.r3.7HG0652410
39.0 HORVU.MOREX.r3.7HG0652600
55.7 HORVU.MOREX.r3.7HG0658370
69.4 HORVU.MOREX.r3.7HG0662310
69.5 HORVU.MOREX.r3.7HG0662360
85.0 HORVU.MOREX.r3.7HG0665250
112.1 HORVU.MOREX.r3.7HG0670130
142.4 HORVU.MOREX.r3.7HG0675210
143.1 HORVU.MOREX.r3.7HG0675320
148.9 HORVU.MOREX.r3.7HG0676140
353.0 HORVU.MOREX.r3.7HG0697110
379.1 HORVU.MOREX.r3.7HG0699700
382.4 HORVU.MOREX.r3.7HG0699930
447.3 HORVU.MOREX.r3.7HG0708130
454.1 HORVU.MOREX.r3.7HG0709010
486.1 HORVU.MOREX.r3.7HG0713300 HORVU.MOREX.r3.7HG0713310
492.2 HORVU.MOREX.r3.7HG0714310
494.0 HORVU.MOREX.r3.7HG0714610
533.5 HORVU.MOREX.r3.7HG0720890
545.0 HORVU.MOREX.r3.7HG0722880
555.9 HORVU.MOREX.r3.7HG0725240
578.4 HORVU.MOREX.r3.7HG0730500
587.1 HORVU.MOREX.r3.7HG0733440
587.8 HORVU.MOREX.r3.7HG0733680 HORVU.MOREX.r3.7HG0733690
589.5 HORVU.MOREX.r3.7HG0734220 HORVU.MOREX.r3.7HG0734230
591.7 HORVU.MOREX.r3.7HG0735160
603.3 HORVU.MOREX.r3.7HG0739720
605.2 HORVU.MOREX.r3.7HG0740430
608.0 HORVU.MOREX.r3.7HG0741530
609.5 HORVU.MOREX.r3.7HG0742540
610.8 HORVU.MOREX.r3.7HG0743040
613.7 HORVU.MOREX.r3.7HG0744420 HORVU.MOREX.r3.7HG0744430
621.3 HORVU.MOREX.r3.7HG0748340
